# Pyruvate Carboxylase in Macrophages Aggravates Atherosclerosis by Regulating Metabolism Reprogramming to Promote Inflammatory Responses Through the Hypoxia‐Inducible Factor‐1 Signaling Pathway

**DOI:** 10.1002/advs.202417128

**Published:** 2025-05-20

**Authors:** Ling‐Na Zhao, Rui‐Ling Wang, Ran‐Xin Liu, Meng‐Ru Zheng, Li Zhao, Bao‐Feng Li, Jia‐Le Li, De‐Shen Liu, Xiao‐Xia He, Qin‐Bao Peng, Kai Li, Tian‐Xiao Lin, Ying‐Ying Liu, Sheng‐Ping He, Jun Lu, Shao‐Yi Zheng, Xiu Liu, Fang‐Ze Huang

**Affiliations:** ^1^ Department of Cardiovascular Surgery Nanfang Hospital Southern Medical University Guangzhou 510515 China; ^2^ Department of Orthopaedics General Hospital of Southern Theater Command of PLA The First School of Clinical Medicine Southern Medical University Guangzhou 510010 China; ^3^ Department of Cardiovascular Surgery Zhujiang Hospital Southern Medical University Guangzhou 510280 China; ^4^ Laboratory of Cardiovascular Science Beijing Clinical Research Institute Beijing Friendship Hospital Capital Medical University Beijing 100050 China; ^5^ Department of Pharmacy The First Affiliated Hospital of USTC Division of Life Sciences and Medicine University of Science and Technology of China Hefei 230001 China; ^6^ Institute of Pediatrics Guangzhou Women and Children's Medical Centre Guangzhou Medical University Guangzhou 511400 China

**Keywords:** atherosclerosis, macrophages, metabolism reprogramming, mitochondria, pyruvate carboxylase

## Abstract

Atherosclerosis (AS) is a major cause of cardiovascular diseases, driven by chronic inflammation and macrophage polarization toward a proinflammatory phenotype. Pyruvate carboxylase (PC), a mitochondrial enzyme involved in glucose metabolism, is implicated in various metabolic disorders; however, its role in AS remains unclear. This study aims to investigate the role and mechanism of PC on macrophages in AS. PC is upregulated in macrophages of humans and mice with AS. Myeloid cell‐specific PC knockout mice are generated to investigate the effects of PC deletion on atherosclerotic plaque formation. Myeloid cell‐specific PC deficiency mitigates high‐fat diet‐induced atherosclerotic lesions in apolipoprotein E knockout mice and mice injected with adeno‐associated virus‐*PCSK9^DY^*. PC deletion enhances mitochondrial respiration and reduces glycolytic activity, thereby reducing reactive oxygen species overproduction and mitochondrial damage in macrophages. PC activates the hypoxia‐inducible factor‐1 (HIF‐1) signaling pathway through macrophage metabolic reprogramming. PC induces nuclear translocation of HIF‐1α in atherosclerotic aortic roots by preventing HIF‐1α from proteasome degradation. HIF‐1α stabilizer reverses the anti‐inflammatory effect of macrophage‐PC ablation in atherogenesis; however, inhibiting HIF‐1α suppresses the proinflammatory macrophage phenotype induced by PC overexpression. This study indicates that macrophage PC aggravates AS through macrophage metabolic reprogramming, promoting inflammatory responses in macrophages through the HIF‐1 signaling pathway.

## Graphical abstract

This study investigates the role of macrophage pyruvate carboxylase (PC) in atherosclerosis (AS) demonstrating that PC upregulation in macrophages promotes metabolism reprogramming to enhance inflammatory responses via the HIF‐1 signaling pathway. Deletion of PC in macrophages reduces atherosclerotic plaque formation by enhancing mitochondrial respiration and modulating macrophage phenotype, highlighting PC as a potential therapeutic target for AS.

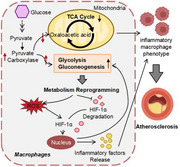

## 1 Introduction

Atherosclerosis (AS) is a leading cause of severe cardiovascular diseases, often resulting in significant global morbidity and mortality.^[^ ^1^ ^]^ Inflammation, dyslipidemia, and oxidative stress predominantly account for the initiation and progression of AS.^[^ ^2^ ^]^ Macrophages primarily contribute to pathologically activating various cell types involved.^[^ ^3^ ^]^ They significantly influence all stages of AS, facilitating the uptake of oxidized low‐density lipoprotein (oxLDL), resulting in lipid accumulation and foam cell formation at early stages, further aggravating atherosclerotic plaque necrotic core at advanced stages.^[^ ^4^ ^]^ The inflammatory macrophage phenotype is crucial in AS. It continuously secretes proinflammatory cytokines, leading to a chronic inflammatory response during AS progression.^[^ ^5^, ^6^ ^]^ Furthermore, accumulating evidence has emphasized the significance of macrophage phenotype modulation in treating AS.

Recent studies have focused on the role of macrophage metabolism in chronic inflammatory diseases. Metabolic changes are vital for determining the function of macrophages in different states because macrophages depend on numerous bioenergetic and biosynthetic processes for phenotypic transition.^[^ ^7^ ^]^ Notably, the primary energy source for inflammatory macrophages is glycolysis, and anti‐inflammatory macrophages use the tricarboxylic acid (TCA) cycle and oxidative phosphorylation (OXPHOS) for energy production.^[^ ^8^ ^]^ Inflammatory macrophages are induced by high levels of reactive oxygen species (ROS), which are associated with increased glycolytic activity and mitochondrial dysfunction.^[^ ^7^, ^9^ ^]^ In contrast, mitochondria in anti‐inflammatory macrophages produce more Adenosine Triphosphate (ATP) through OXPHOS but generate fewer ROS than inflammatory macrophages.^[^ ^8^, ^10^ ^]^ This underscores an essential role of glucose metabolism and mitochondrial function in macrophage phenotype modulation.

Pyruvate carboxylase (PC) is a pivotal mitochondrial enzyme that converts pyruvate into oxaloacetate.^[^ ^11^ ^]^ This conversion supplements the TCA cycle intermediate in mitochondria or promotes gluconeogenesis in the cytoplasm.^[^ ^12^ ^]^ Thus, PC is associated with the TCA cycle and gluconeogenesis.^[^ ^13^ ^]^ Reportedly, the metabolic intermediates produced by PC may influence oxidative stress in the mitochondria. Additionally, PC has been associated with various types of tumors and metabolic disorders, such as diabetes mellitus.^[^ ^14^, ^15^, ^16^ ^]^ However, PC's role in metabolism and mitochondrial function in AS remains unclear. Therefore, we aimed to investigate the effects of PC on AS and explore its therapeutic potential in atherosclerotic treatment.

## 2 Results

### 2.1 PC was Upregulated in Macrophages of Humans and Mice with Atherosclerosis

**Figure 1:**
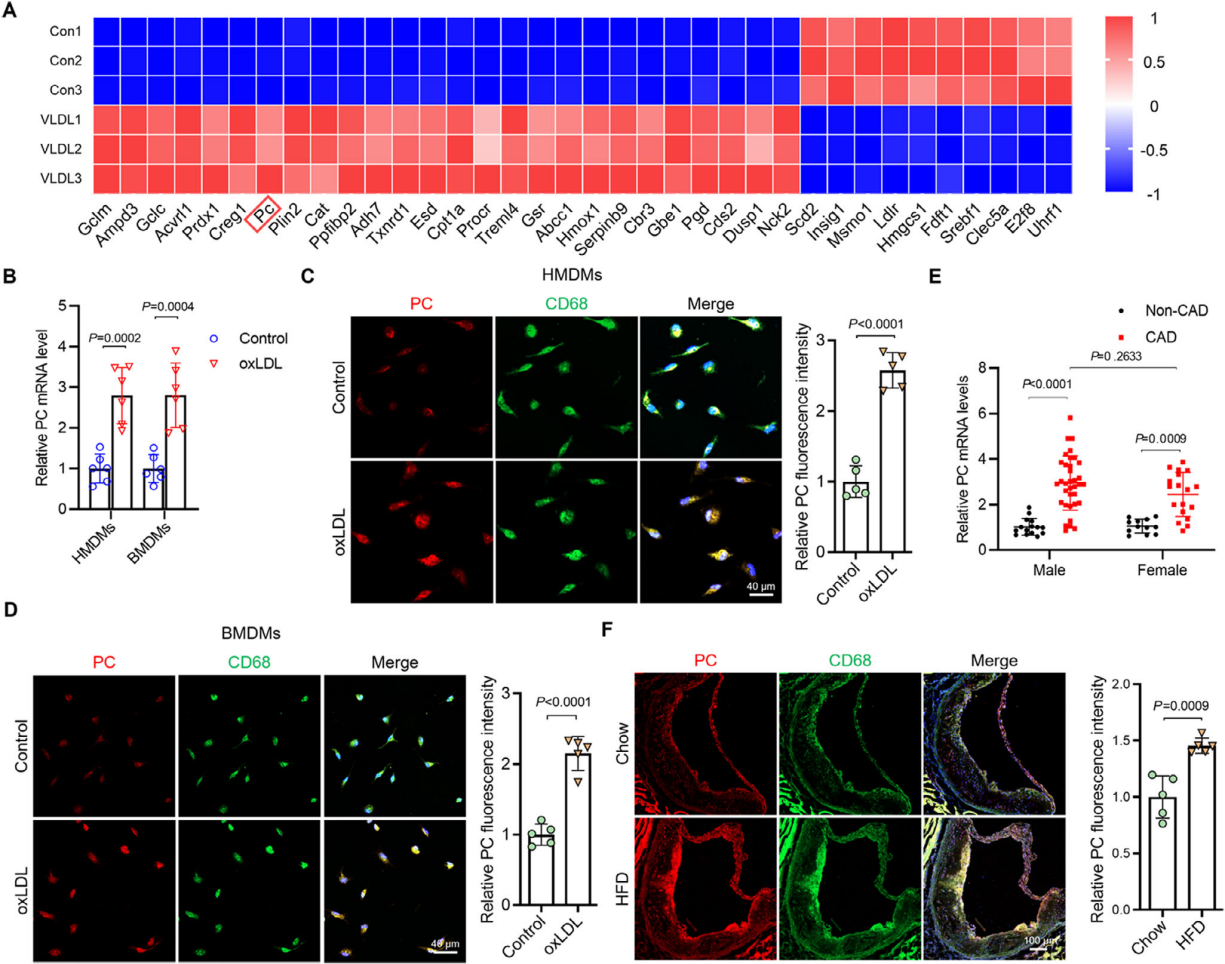
PC was upregulated in macrophages of humans and mice with atherosclerosis. A) Heatmap of differential genes from the publicly available RNA sequencing data (GSE203250) of RAW 264.7 macrophages treated with VLDL‐sized emulsion particles or vehicle (*n* = 3). B) PC mRNA levels in HMDMs and BMDMs treated with oxidized low‐density lipoproteins (oxLDL, 80 µg mL^−1^) for 24 h (*n* = 6). C,D) Cells were treated with oxLDL (80 µg mL^−1^) for 24 h (*n* = 5). Representative immunofluorescence images of PC and CD68 in C) HMDMs and D) BMDMs. Scale bars: 40 µm. E) PC mRNA levels in the PBMCs of healthy participants (non‐CAD) (*n* = 27; male vs female: 15:12) and patients with CAD (CAD) (*n* = 53; male vs female: 35:18). F) Representative immunofluorescence images of PC and CD68 in the plaques of aortic sinuses from *ApoE^−/−^* male mice fed with chow or HFD (*n* = 5). The fluorescence intensity of PC was quantified. Scale bars: 100 µm. Data are presented as means ± SD. B–D,F) Unpaired two‐tailed *t‐test* was used. E) Two‐way analysis of variance with Tukey's correction was used. VLDL, very low‐density lipoprotein; HMDM, human monocyte‐derived macrophage; BMDM, bone marrow‐derived macrophage; CAD, coronary artery diseases; PBMC, peripheral blood mononuclear cell; HFD, high‐fat diet; PC, pyruvate carboxylase.

We reanalyzed the publicly available RNA‐sequencing data of Raw 264.7 macrophages treated with very low‐density lipoprotein (VLDL)‐sized emulsion particles (GSE203250). We observed that PC was significantly upregulated in the VLDL‐treated group (**Figure** **1**A; and Figure S1A, Supporting Information). Similarly, PC expression was elevated in human monocyte‐derived macrophages (HMDMs) and mouse bone marrow‐derived macrophages (BMDMs) after oxLDL treatment (Figure 1B–D). Next, we isolated peripheral blood mononuclear cells (PBMCs) from healthy participants and patients with coronary artery disease (CAD) and measured PC mRNA levels to further clarify PC's expression in macrophages. PC mRNA levels were significantly higher in CAD patients than in controls, regardless of the gender (Figure 1E). These results suggest a potential association between PC expression in macrophages and AS. Furthermore, we induced an atherosclerotic mouse model by feeding apolipoprotein E knockout (*ApoE^−/−^*) mice with a high‐fat diet (HFD) for 12 weeks and examining PC expression. We measured the mRNA levels of PC in the aortic smooth muscle cells (MASMCs) and aortic endothelial cells (MAECs) isolated from *ApoE^−/−^* mice fed with a chow diet or HFD. Notably, no significant difference of PC levels was observed in both MASMCs and MAECs from atherosclerotic mice (Figure S1B, Supporting Information). Furthermore, PC was found to accumulate in the plaques of the aortic sinuses in atherosclerotic mice, where macrophages were located (Figure 1F). The enzyme activity of PC was also upregulated in atherosclerotic pathology (Figure S1C, S1D, Supporting Information), suggesting the important role of PC in macrophages in the progression of AS.

### 2.2 Myeloid Cell‐Specific PC Deficiency Mitigated HFD‐Induced Atherosclerotic Lesions

**Figure 2:**
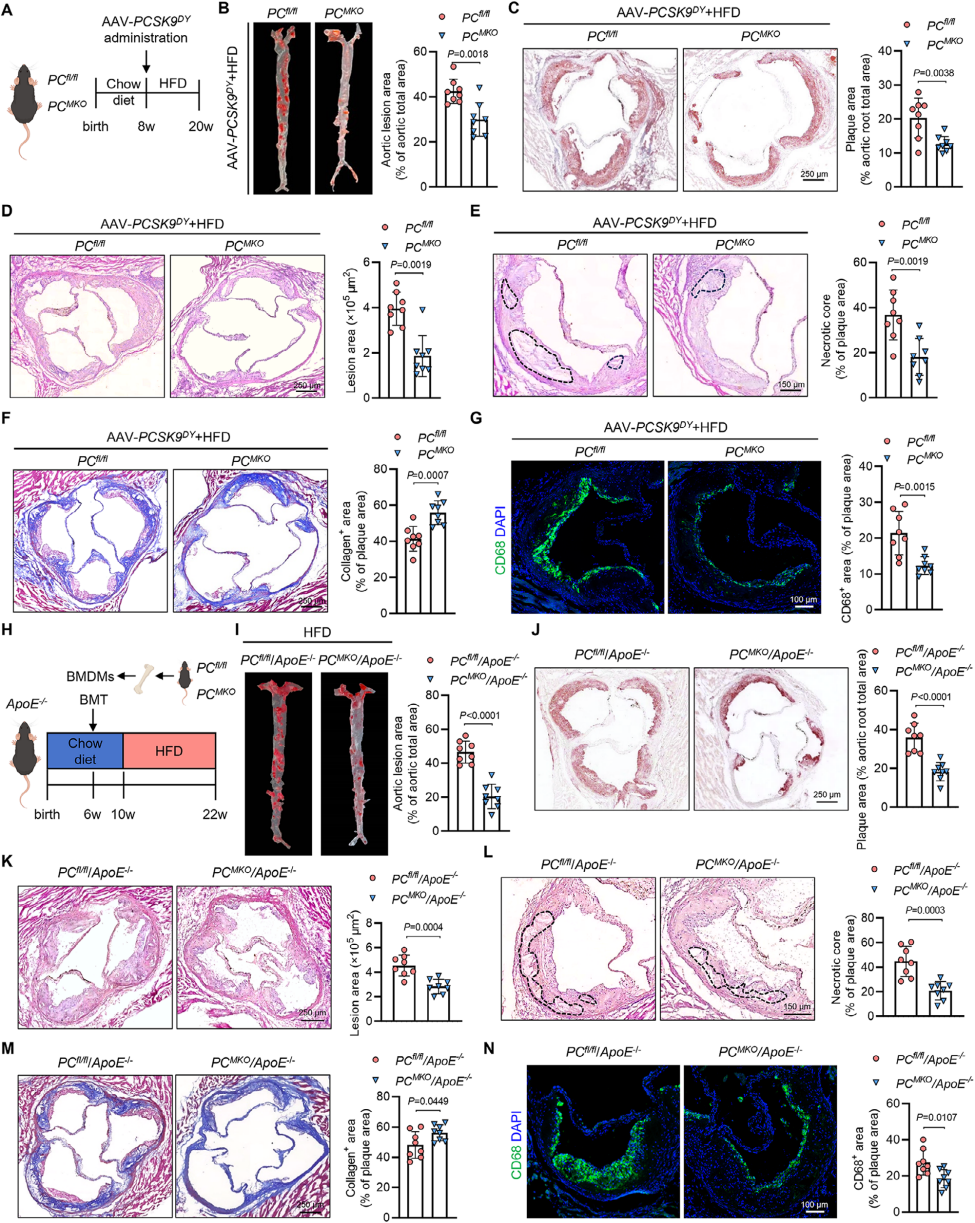
PC deficiency in macrophages mitigated atherosclerosis in AAV‐*PCSK9^DY^*‐induced mice and chimeric *ApoE^−/−^* mice. A) Male *PC^fl/fl^* and *PC^MKO^* mice were administered with AAV‐*PCSK9^DY^* followed by 12 weeks of chow diet or HFD feeding. B) Representative images and quantification of the Oil Red O‐stained aortas from *PC^fl/fl^* and *PC^MKO^* mice administered with AAV‐*PCSK9^DY^* and fed with a 12‐week HFD (*n* = 8). C) Representative images and quantification of the Oil Red O‐stained aortic root sections (*n* = 8). Scale bars: 250 µm. D,E) Representative images of HE staining in aortic root sections from *PC^fl/fl^* and *PC^MKO^* mice administered with AAV‐*PCSK9^DY^* and fed with a 12‐week HFD (*n* = 8). Lesion area D) and necrotic core area E) of the aortic root were quantified. Scale bars for (D): 250 µm; Scale bars for (E): 150 µm. F) Representative images and collagen quantification of the aortic roots stained with Masson staining (*n* = 8). Scale bars: 250 µm. G) Representative immunofluorescence images of the macrophage marker CD68 in the aortic root (*n* = 8). Scale bars: 100 µm. H) Procedure of conducting bone marrow transplantation in male *ApoE^−/^* ^−^ mice. I) Representative images and quantification of the Oil Red O‐stained aortas from chimeric *ApoE^−/^* ^−^ mice that underwent bone marrow transplantation with bone marrow from *PC^fl/fl^* or *PC^MKO^* mice after a 12‐week HFD (*n* = 8). J) Representative images and quantification of the Oil Red O‐stained aortic root sections (*n* = 8). Scale bars: 250 µm. K,L) Representative images of HE staining in aortic root sections from chimeric *ApoE^−/^* ^−^ mice receiving bone marrow from *PC^fl/fl^* or *PC^MKO^* mice after a 12‐week HFD (*n* = 8). Lesion area K) and necrotic core area L) of the aortic root were quantified. Scale bars for (K): 250 µm; Scale bars for (L): 150 µm. M) Representative images and collagen quantification of the aortic roots stained with Masson staining (*n* = 8). Scale bars: 250 µm. N) Representative immunofluorescence images of the macrophage marker CD68 in the aortic root (*n* = 8). Scale bars: 100 µm. Data are presented as means ± SD. B,C,E–G,I–N Unpaired two‐tailed *t‐test* was used. D) Mann–Whitney U test with the exact method was used. AAV, adeno‐associated virus; HFD, high‐fat diet; PC, pyruvate carboxylase; HE, hematoxylin and eosin; bone marrow‐derived macrophage; BMT, bone marrow transplantation.

We generated myeloid cell‐specific PC knockout mice (*Lyz2‐Cre‐PC^flox^*) to determine the role of PC in macrophages in AS, hereafter referred to as *PC^MKO^* mice (Figure S2A ,S2B, Supporting Information). The null allele was verified in the aortic roots of *PC^MKO^* mice and the BMDMs isolated from *PC^MKO^* mice using western blotting and immunofluorescence (Figure S2C–S2E, Supporting Information). An adeno‐associated virus‐*PCSK9^DY^* (AAV‐*PCSK9^DY^*) was administered to the mice to induce AS, followed by HFD feeding for 12 weeks (**Figure** **2**A). Our previous study confirmed that the *PCSK9^DY^* mutation induces degradation of the hepatic low‐density lipoprotein receptor (LDLR) and thus mimics the effect of *LDLR* knockout in mice.^[^ ^17^, ^18^ ^]^ Consistently, the LDLR protein was barely detected in the livers of mice injected with AAV‐*PCSK9^DY^* in our study (Figure S2F, Supporting Information). Notably, myeloid cell‐specific PC deficiency hardly affected the body weight or serum lipids of mice fed either a chow diet or HFD (Figure S2G–S2K, Supporting Information). However, after feeding with HFD, *PC^MKO^* mice showed less atherogenesis compared to *PC^fl/fl^* mice. Additionally, smaller plaque areas were observed in the aorta and aortic roots, detected via Oil Red O staining, whereas mice fed with a chow diet developed little atherogenesis (Figure 2B,C; and Figure S2L, S2M, Supporting Information). Moreover, the lesion and necrotic core areas of the aortic roots from *PC^MKO^* mice were reduced, while the collagen area was increased (Figure 2F). Consistent with the results in aorta and aortic root, *PC^MKO^* mice showed less plaque and lesion areas in carotid arteries compared to *PC^fl/fl^* mice, suggesting the protective effect of PC deletion on atherogenesis (Figure S2N, S2O, Supporting Information). Few CD68^+^ macrophages infiltrated the plaques of aortic roots from *PC^MKO^* mice (Figure 2G). However, the number of dead and proliferating cells in the plaques of *PC^MKO^* and *PC^fl/fl^* mice did not differ (Figure S2P, S2Q, Supporting Information).

Furthermore, we performed bone marrow transplantation (BMT) to generate PC myeloid cell‐specific deletion in an *ApoE^−/−^* background. Briefly, the bone marrow from *PC^MKO^* and *PC^fl/fl^* mice was extracted and administered to *ApoE^−/−^* male mice that were lethally irradiated for marrow cell clearance (Figure 2H). Polymerase chain reaction (PCR) of genomic DNA from peripheral blood was performed to determine whether bone marrow chimeras were successfully generated (Figure S3A, Supporting Information). Additionally, immunofluorescence staining confirmed that PC was effectively deleted in BMDMs from *ApoE^−/−^* mice (Figure S3B, Supporting Information). No significant differences in body weight or serum lipid levels were observed between *PC^MKO^/ApoE^−/−^* and *PC^fl/fl^/ApoE^−/−^* mice (Figure S3C–S3G, Supporting Information). Consistent with *PC^MKO^* mice with AAV‐*PCSK9^DY^* administration, *PC^MKO^/ApoE^−/−^* mice showed reduced plaque areas in the aorta and aortic roots (Figure 2I,J; and Figure S3H, S3I, Supporting Information). Smaller lesions and necrotic areas but larger collagen areas in the aortic roots were observed in *PC^MKO^/ApoE^−/−^* mice (Figure 2K–M). The area of CD68^+^ macrophage infiltration in the aortic roots of *PC^MKO^/ApoE^−/−^* mice decreased (Figure 2N); however, dead cells and proliferating cells in the plaques did not differ significantly (Figure S3J, S3K, Supporting Information).

### 2.3 PC Promoted an Inflammatory Macrophage Phenotype and Foam Cell Formation

**Figure 3:**
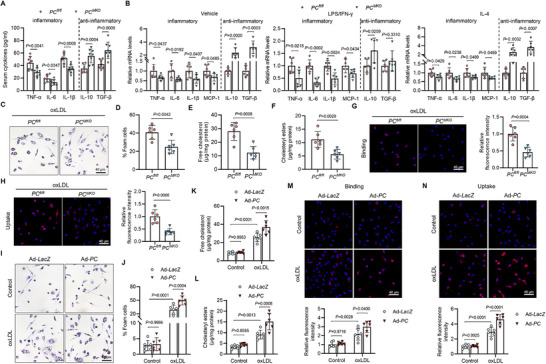
PC promoted an inflammatory macrophage phenotype and foam cell formation. A) Serum levels of TNF‐α, IL‐6, IL‐1β, IL‐10, and TGF‐β in *PC^fl/fl^* and *PC^MKO^* mice administered with AAV‐*PCSK9^DY^* and fed with a 12‐week HFD (*n* = 8). B) Quantification of selected mRNA in BMDMs from *PC^fl/fl^* and *PC^MKO^* mice treated differently (*n* = 6). C–H) BMDMs from male *PC^fl/fl^* and *PC^MKO^* mice were treated with oxLDL (80 µg mL^−1^) (*n* = 6). C) Representative images of foam cells in *PC^fl/fl^* and *PC^MKO^* BMDMs. Scale bars: 40 µm. Quantification of the ratio of foam cells D), unesterified cholesterol E), and cholesteryl ester F) in BMDMs from *PC^fl/fl^* and *PC^MKO^* mice. Representative images of binding G) and uptake H) of Dil labeled oxLDL (Dil‐oxLDL) in BMDMs. Scale bars: 40 µm. I–N) Primary BMDMs transfected with Ad‐*LacZ* or Ad‐*PC* were treated with oxLDL (80 µg mL^−1^) or PBS (*n* = 6). I) Representative images of foam cells in BMDMs transfected with Ad‐*LacZ* and Ad‐*PC*. Scale bars: 40 µm. Quantification of the ratio of foam cells J), unesterified cholesterol K), and cholesteryl ester L) in BMDMs. Representative images of binding M) and uptake N) of Dil‐oxLDL in BMDMs. Scale bars: 40 µm. Data are presented as means ± SD. A,B,D–H) Unpaired two‐tailed *t*‐test was used. J–N) Two‐way analysis of variance with Tukey's correction was used. LPS, lipopolysaccharide; IFN‐γ, interferon‐γ; IL, interleukin; oxLDL, oxidized low‐density lipoproteins; BMDM, bone marrow‐derived macrophage; PC, pyruvate carboxylase.

In the serum of atherosclerotic *PC^MKO^* mice, inflammatory cytokine levels, such as tumor necrosis factor‐Alpha (TNF‐α), interleukin (IL)‐6, and IL‐1β, were reduced; however, levels of anti‐inflammatory cytokines, IL‐10 and transforming growth factor‐beta (TGF‐β) were elevated (**Figure** **3**A). This observation suggests that PC may affect macrophage polarization during AS. We treated BMDMs from *PC^MKO^* and *PC^fl/fl^* mice with vehicle, lipopolysaccharide/interferon‐c (LPS/IFN‐γ), or IL‐4 and examined the cytokine level to verify the role of PC in macrophages. PC deletion resulted in a reduction of inflammatory cytokines, including TNF‐α, IL‐6, IL‐1β, and monocyte chemoattractant protein‐1(MCP‐1), in all groups, particularly in the LPS/IFN‐γ‐treated group. However, PC deficiency enhanced the expression of anti‐inflammatory cytokines, such as IL‐10 and TGF‐β, in vehicle‐ and IL‐4‐treated BMDMs (Figure 3B). These findings indicate that PC promotes an inflammatory macrophage phenotype. Furthermore, we investigated functional changes in macrophages. PC deletion reduced foam cell formation and lipid accumulation in BMDMs (Figure 3C–F). In addition, binding and uptake assays revealed that 1,1′‐dioctadecyl‐3,3,3′,3′‐tetramethylindocarbocyanine‐labeled oxidized low‐density lipoprotein (Dil‐oxLDL) binding and uptake were suppressed in BMDMs from *PC^MKO^* mice (Figure 3G,H). To further determine the effect of PC on macrophage function, we treated primary BMDMs extracted from wild‐type C57BL/6J mice with a PC adenovirus (Ad‐*PC*) (Figure S4A, S4B, Supporting Information). PC upregulation induced the secretion of inflammatory factors in macrophages, which suppressed the secretion of anti‐inflammatory factors (Figure S4C, Supporting Information). Furthermore, PC overexpression enhanced foam cell formation and lipid accumulation (Figure 3I–L). Dil‐oxLDL immunofluorescence showed that PC overexpression potentiated the binding and uptake of oxLDL in macrophages (Figure 3M,N). These data show that PC promotes the transformation of macrophages into an inflammatory phenotype, thereby exacerbating foam cell formation.

### 2.4 PC Deletion Reduced Metabolism Reprogramming and Mitochondrial Damage in Macrophages

**Figure 4:**
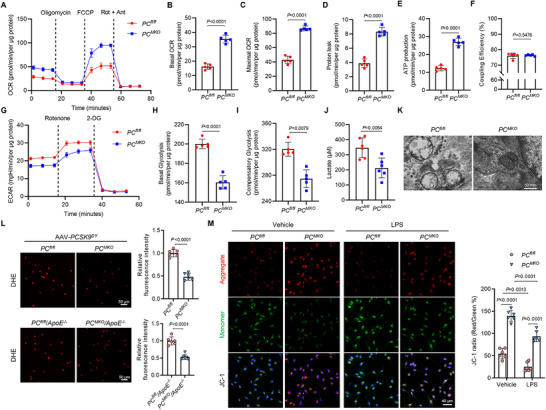
PC deletion reduced metabolic reprogramming and mitochondrial damage in macrophages. A–F) Male *PC^fl/fl^* and *PC^MKO^* mice that were administered with AAV‐*PCSK9^DY^* and fed with a 12‐week HFD were sacrificed. BMDMs were collected for the Seahorse Mito Stress Test (*n* = 5). Normalized OCR tracing A), basal OCR B), maximal OCR C), proton leak D), ATP production E), and coupling efficiency F) in *PC^fl/fl^* and *PC^MKO^* BMDMs. G–I) *PC^fl/fl^* and *PC^MKO^* mice that were administered with AAV‐*PCSK9^DY^* and fed with a 12‐week HFD were sacrificed. BMDMs from *PC^fl/fl^* and *PC^MKO^* mice administered with AAV‐*PCSK9^DY^* and fed with a 12‐week HFD were collected for the Seahorse Glycolytic Rate Assay (*n* = 5). Normalized ECAR tracing G), basal glycolysis H), and compensatory glycolysis I) in *PC^fl/fl^* and *PC^MKO^* BMDMs. J) Lactate levels in BMDMs from *PC^fl/fl^* and *PC^MKO^* mice administered with AAV‐*PCSK9^DY^* and fed with a 12‐week HFD (*n* = 6). K) Mitochondrial morphology observed via transmission electron microscopy. Magnification ×15, scale bar: 500 nm. L) *PC^fl/fl^* and *PC^MKO^* mice that were administered with AAV‐*PCSK9^DY^* and chimeric *ApoE^−/^* ^−^ mice that underwent bone marrow transplantation from *PC^fl/fl^* or *PC^MKO^* mice were sacrificed to collect BMDMs. Representative images of DHE staining for ROS detection in BMDMs (*n* = 6). Scale bars: 50 µm. M) Representative JC‐1 staining and relative JC‐1 signal intensity in *PC^fl/fl^* and *PC^MKO^* BMDMs treated with vehicle or LPS (100 ng mL^−1^, 12 h) (*n* = 6). Scale bars: 40 µm. Data are presented as means ± SD. B–E,H,J,L Unpaired two‐tailed *t*‐test was used. F,I) Mann–Whitney U test with the exact method was used. M) Two‐way analysis of variance with Tukey's correction was used. OCR, oxygen consumption rate; ECAR, extracellular acidification rate; DHE, dihydroethidium; LPS, lipopolysaccharides; BMDM, bone marrow‐derived macrophage; HFD, high‐fat diet; PC, pyruvate carboxylase.

PC is involved in the TCA cycle and gluconeogenesis by catalyzing pyruvate carboxylation to form oxaloacetate.^[^ ^11^ ^]^ We investigated whether PC influences macrophages by interfering with mitochondrial function during metabolism considering that PC is important in glucose metabolism. Seahorse extracellular flux analysis revealed that basal and maximal oxygen consumption rates (OCRs) in *PC^MKO^* BMDMs were elevated, including proton leakage and ATP production (**Figure** **4**A–F), indicating that PC ablation enhanced mitochondrial respiration. Furthermore, extracellular acidification rate analysis (ECAR) showed that glycolysis and glycolytic capacity were reduced in *PC^MKO^* BMDMs, along with reduced lactate production (Figure 4G–J). Collectively, these results suggest that PC induces a metabolic switch from mitochondrial OXPHOS to glycolysis, as OXPHOS were not the primary energy sources in macrophages.

We further observed the mitochondrial structure using transmission electron microscopy (TEM) to investigate the effect of PC on mitochondria. Fewer swollen mitochondria with degenerated cristae were observed in *PC^MKO^* BMDMs compared to *PC^fl/fl^* BMDMs (Figure 4K). Next, we examined ROS production in macrophages from atherosclerotic mice using in situ dihydroethidium staining. We found that ROS generation was attenuated in BMDMs isolated from *PC^MKO^* and *PC^MKO^/ApoE^−/−^* mice (Figure 4L), indicating a protective effect of PC deletion on mitochondria. To analyze early‐stage mitochondrial apoptosis, we used JC‐1 staining to assess mitochondrial membrane potential. LPS aggravated mitochondrial apoptosis in macrophages, which was attenuated by PC deficiency (Figure 4M). In summary, these results suggest that PC plays a role in in regulating mitochondrial function and metabolism in macrophages under pathological conditions.

### 2.5 PC Activated the Hypoxia‐Inducible Factor‐1 (HIF‐1) Signaling Pathway by Initiating Metabolism Reprogramming in Macrophages

**Figure 5:**
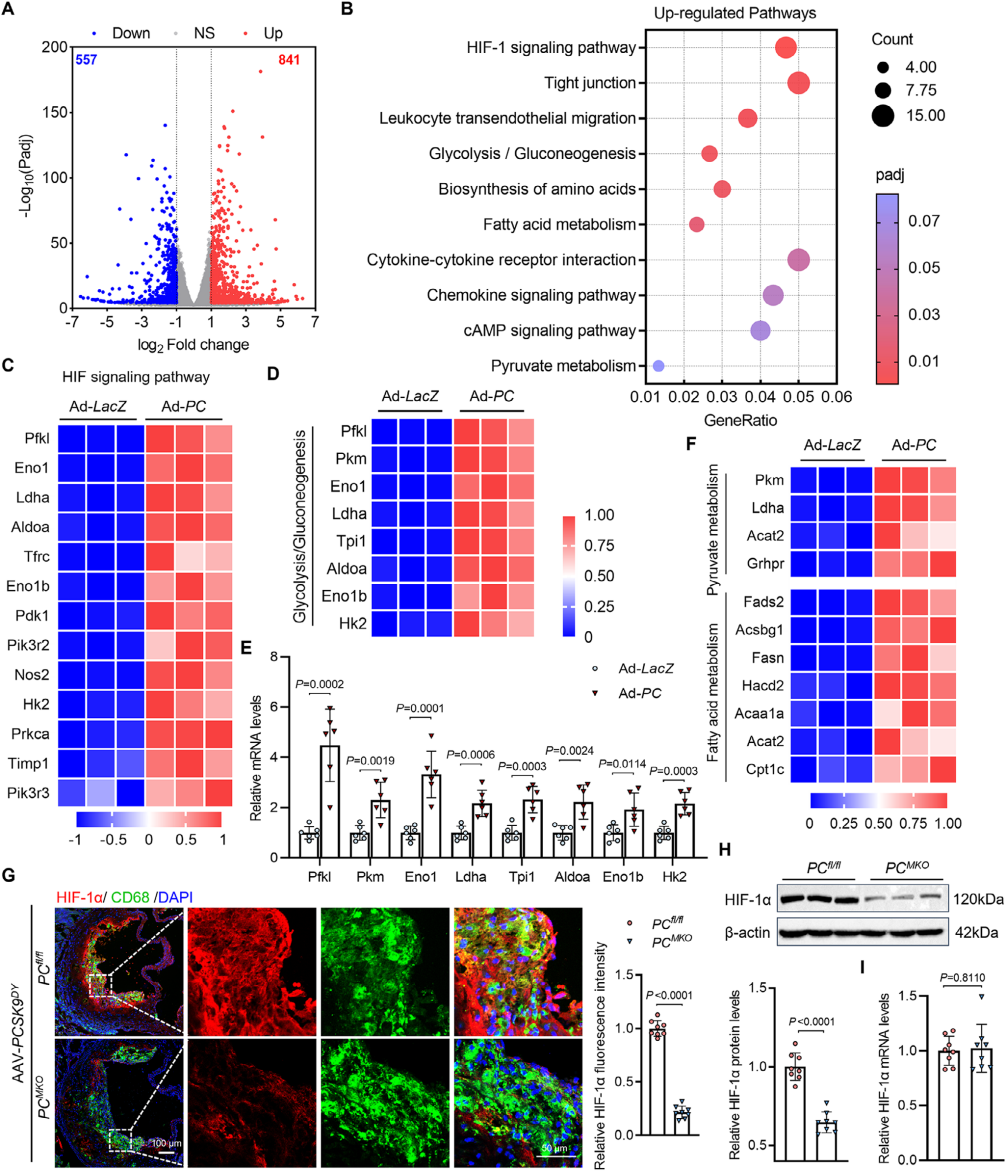
PC activated the HIF‐1 signaling pathway by initiating metabolic reprogramming in macrophages. A–D,F) RNA‐sequencing analysis of BMDMs transfected with Ad‐*LacZ* and Ad‐*PC* (*n* = 3). A) Volcano plot representing the comparison of gene expression in BMDMs transfected with Ad‐*LacZ* and Ad‐*PC*. Differentially expressed genes were defined as those with a greater than twofold change and an adjusted *P* value (Padj) <0.05. Upregulated and downregulated differentially expressed genes are colored in red and blue, respectively. B) KEGG enrichment analysis of RNA sequencing data. C) Heatmap showing the comparison of differentially expressed genes in the HIF‐1 signaling pathway. D) Heatmap revealing the comparison of differentially expressed genes associated with glycolysis and gluconeogenesis. E) Quantification of mRNA levels of genes involved in the HIF‐1 signaling pathway in BMDMs transfected with Ad‐*LacZ* and Ad‐*PC* (*n* = 6). F) Heatmap showing the comparison of differentially expressed genes in pyruvate and fatty acid metabolism. G–I) *PC^fl/fl^* and *PC^MKO^* mice that were administered with AAV‐*PCSK9^DY^* and fed with a 12‐week HFD were sacrificed, and BMDMs were collected for examination (*n* = 8). G) Representative immunofluorescence images of HIF‐1α and CD68 in the plaques of aortic sinuses from *PC^fl/fl^* and *PC^MKO^* mice. Scale bars: 100 µm, enlarged: 50 µm. H) Representative HIF‐1α western blotting and protein quantification in *PC^fl/fl^* and *PC^MKO^* BMDMs. I) HIF‐1α mRNA levels in *PC^fl/fl^* and *PC^MKO^* BMDMs. Data are presented as means ± SD. E,G–I) Unpaired two‐tailed *t*‐test was used. AAV, adeno‐associated virus; BMDM, bone marrow‐derived macrophage; HIF‐1, hypoxia‐inducible factor 1; PC, pyruvate carboxylase; KEGG, Kyoto Encyclopedia of Genes and Genomes; HFD, high‐fat diet.

RNA sequencing analysis of BMDMs transfected with Ad‐*LacZ* and Ad‐*PC* was performed to determine the mechanisms by which PC influences the cellular metabolism and function in macrophages (Figure S5A, Supporting Information). Bioinformatics analysis revealed that 841 genes were upregulated, and 557 genes were downregulated in BMDMs overexpressing PC (**Figure** **5**A). Differentially expressed genes were predominantly enriched in metabolism‐related pathways (Figure S5B, S5C, Supporting Information). The upregulated genes of BMDMs transfected with Ad‐*PC* were notably enriched in the HIF‐1 signaling pathway, as well as in metabolic pathways, such as glycolysis/gluconeogenesis, biosynthesis of amino acids, fatty acid metabolism, and pyruvate metabolism (Figure 5B,C). We then analyzed the genes involved in glycolysis/gluconeogenesis that were upregulated in PC‐overexpressing BMDMs. Genes such as *Prk1*, *Pkm*, *Eno1*, *Ldha*, *Tpi1*, *Aldoa*, *Enob1*, and *Hk2* were differentially expressed, as confirmed by PCR (Figure 5D,E). Additionally, some significantly upregulated genes, including *Prk1*, *Eno1*, *Ldha*, *Aldoa*, and *Hk2*, are involved in the HIF‐1 signaling pathway. Furthermore, pyruvate and fatty acid metabolism were activated, and the same differentially expressed genes were found to be upregulated (Figure 5F). We also examined the expression of mitochondrial function‐related genes and found PC overexpression suppressed the expression coding enzymes in TCA cycle and electron transport chain (ETC) complexes, suggesting that PC reduced OXPHOS in macrophages (Figure S5D, S5E, Supporting Information). Interestingly, PC also modulated the expression of scavenger receptors, such as CD36 and ABCG1, with PC overexpression upregulating CD36 and downregulating ABCG1, while SR‐A1 expression was barely affected (Figure S5F, S5G, Supporting Information). This explained the results above that PC might enhance foam cell formation and lipid accumulation by influencing scavenger receptors in macrophages.

We next examined whether the featured factor, HIF‐1α, was linked to the altered metabolism in macrophages caused by PC, as the HIF‐1 pathway was activated by PC. In atherosclerotic *PC^MKO^* mice, HIF‐1α expression was reduced in the macrophage‐infiltrating regions of the aortic roots, with less translocation of HIF‐1α to the nucleus of macrophages (Figure 5G). Next, we examined HIF‐1α expression in *PC^MKO^* BMDMs and found that HIF‐1α protein was reduced under PC deletion (Figure 5H). However, the mRNA level of HIF‐1α was not significantly different between *PC^MKO^* and *PC^fl/fl^* BMDMs (Figure 5I), suggesting that PC might not affect HIF‐1α expression by disturbing its transcription.

### 2.6 PC Upregulated HIF‐1α by Preventing Its Proteasome Degradation in Macrophages

**Figure 6:**
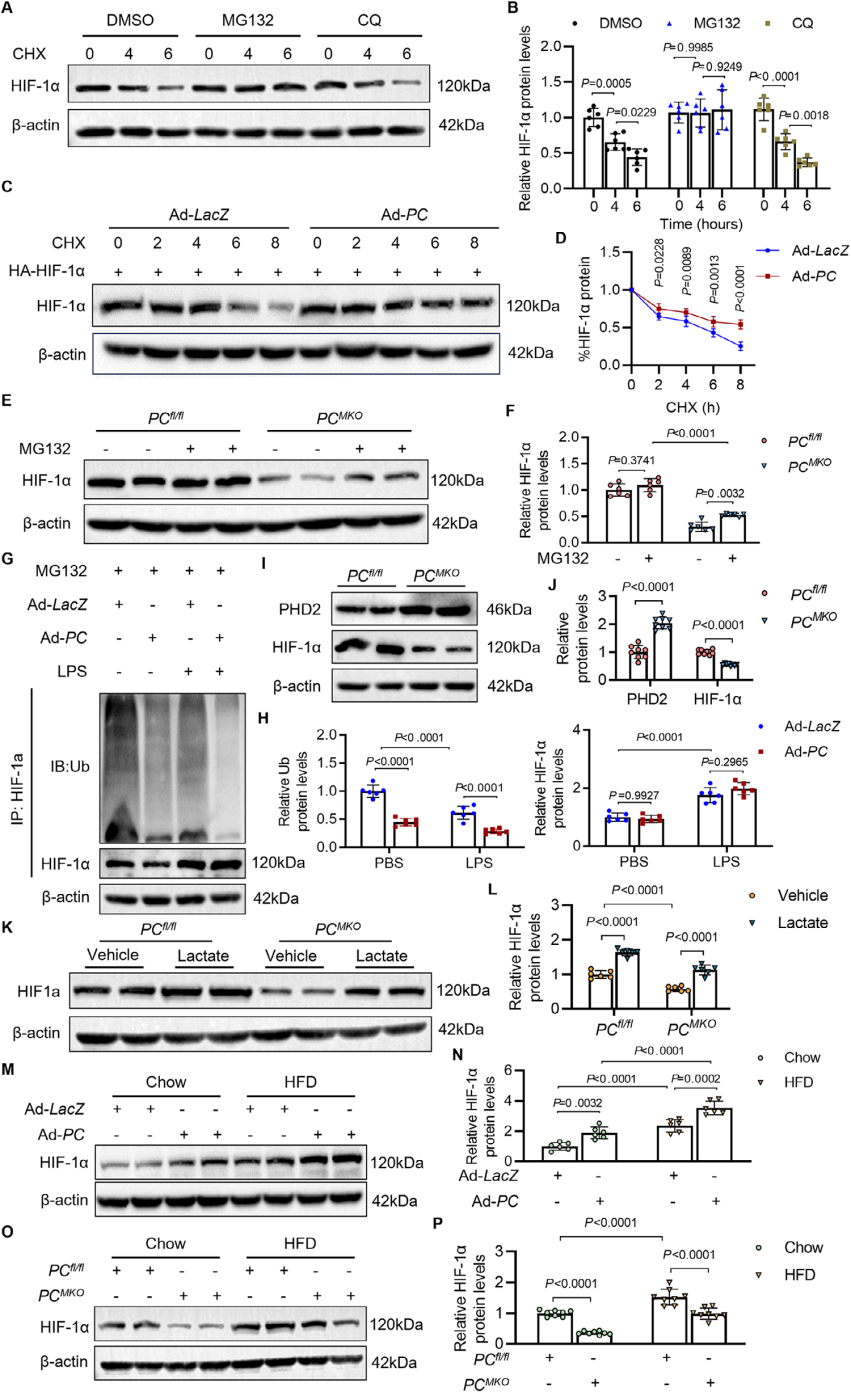
PC upregulated HIF‐1α by preventing its proteasome degradation in macrophages. A,B) BMDMs were isolated from male *PC^MKO^* mice and treated with cycloheximide (CHX, 50 µg mL^−1^), along with MG132 (10 µmol L^−1^) or chloroquine (CQ, 20 µmol L^−1^) at different timepoints (*n* = 6). Representative western blotting A) and quantification B) of HIF‐1α protein in BMDMs with different treatments. C,D) Mouse primary BMDMs were transfected with HA‐HIF‐1α plasmids and Ad‐*LacZ* or Ad‐*PC*, followed by CHX treatment at the indicated time (*n* = 5). HIF‐1α expression C) and quantification D) in BMDMs at different timepoints are presented. E,F) BMDMs were isolated from male *PC^fl/fl^* and *PC^MKO^* mice and treated with DMSO or MG132 (10 µmol L^−1^) (*n* = 6). Representative western blotting E) and quantification F) of HIF‐1α protein in BMDMs with different treatments. G,H) Mouse primary BMDMs were treated with MG132 (10 µmol L^−1^) and transfected with Ad‐*LacZ* or Ad‐*PC*, followed by LPS (100 ng mL^−1^) stimulation for 12 h (*n* = 6). Lysates were immunoprecipitated using the HIF‐1α antibody and immunoblotted with antibodies against the indicated proteins. Representative western blotting G) and quantification H) of indicated proteins in BMDMs with different treatments. I,J) BMDMs were isolated from *PC^fl/fl^* and *PC^MKO^* mice fed with HFD (*n* = 8). Selected proteins expression I) and quantification J) in BMDMs are presented. K,L) BMDMs were isolated from male *PC^fl/fl^* and *PC^MKO^* mice and treated with lactate (5 mm) for 12 h (*n* = 6). Representative western blotting K) and quantification L) of HIF‐1α protein in BMDMs with different treatments. M,N) BMDMs from male *ApoE^−/‐^* mice fed with a chow diet or HFD were collected and transfected with Ad‐*LacZ* or Ad‐*PC* (*n* = 6). HIF‐1α expression M) and quantification N) in BMDMs are presented. O,P) BMDMs from male *PC^fl/fl^* and *PC^MKO^* mice administrated with AAV‐*PCSK9^DY^* and fed with a chow diet or HFD were collected (*n* = 8). HIF‐1α expression O) and quantification P) in BMDMs are presented. Data are presented as means ± SD. B) One‐way analysis of variance (ANOVA) with Tukey's correction was used. D) Two‐way ANOVA with Bonferroni's multiple comparisons test was used. F,H,L,N,P) Two‐way ANOVA with Tukey's correction was used. J) Unpaired two‐tailed *t*‐test was used. DMSO, dimethyl sulfoxide; CHX, cycloheximide; CQ chloroquine; HFD, high‐fat diet; BMDM, bone marrow‐derived macrophage; HIF‐1, hypoxia‐inducible factor 1; PC, pyruvate carboxylase.

We next assessed whether PC affected HIF‐1α protein stability, given that PC barely altered the transcription level of HIF‐1α. We treated *PC^MKO^* BMDMs with the protein synthesis inhibitor cycloheximide (CHX), combined with the lysosomal inhibitor chloroquine (CQ) or the proteasomal inhibitor MG132. Western blot analysis revealed that the loss of HIF‐1α protein induced by CHX could be reversed by MG132 (**Figure** **6**A,B). Furthermore, we transfected BMDMs with Ad–*PC*, with the transfection of HA–HIF‐1α plasmids and incubation of CHX. PC overexpression inhibited the reduced exogenous HIF‐1α expression due to CHX treatment, demonstrating that PC contributed to maintaining HIF‐1α protein stability (Figure 6C,D). In *PC^MKO^* BMDMs, MG132 treatment reversed HIF‐1α protein degradation caused by PC ablation (Figure 6E,F). These data suggest that the positive regulation of HIF‐1α expression by PC is associated with proteasome degradation.

Ubiquitination is an essential part of proteasomal degradation, where the target protein is covalently attached to ubiquitin and thus recognized by the proteasome for degradation. Accordingly, we assessed the ubiquitination level of HIF‐1α in primary BMDMs under different treatments. Immunoprecipitation results showed that LPS increased HIF‐1α expression in macrophages, and PC reduced the ubiquitinated form of HIF‐1α (Figure 6G,H). The stabilization of HIF‐1α depends on the regulation of prolyl hydroxylase domain‐containing enzymes (PHD), especially PHD2.^[^ ^19^ ^]^ Under normoxic conditions, HIF‐1α is hydroxylated by PHD and thus recognized by Von Hippel–Lindau (VHL) complex for ubiquitination and proteasome degradation.^[^ ^20^ ^]^ However, in hypoxia, PHD activity is inhibited, leading to HIF‐1α stabilization and translocation to the nucleus.^[^ ^21^ ^]^ To verify the mechanism by which PC prevented HIF‐1α ubiquitination, we detected the expression of the key enzyme, PHD2, in BMDMs. PC ablation increased PHD2 levels and decreased HIF‐1α expression, indicating that PC might reduce HIF‐1α ubiquitination and proteasome degradation by inhibiting PHD2 activity (Figure 6I,J). As previous studies have revealed that ROS overproduction and lactate level can influence the expression of HIF‐1α by inhibition of PHDs and VHL function, we also examined the influence of lactate levels on the stabilization of HIF‐1α. Consequently, lactate reversed the reduction of HIF‐1α expression by PC deletion (Figure 6K,L). For further confirmation, we isolated BMDMs from *ApoE^−/−^* mice fed with a chow diet or a HFD and transfected with Ad‐*LacZ* or Ad‐*PC*. The increased HIF‐1α expression was observed in the HFD group, which was enhanced by the overexpression of PC in macrophages (Figure 6M,N). Moreover, PC deletion reduced the expression of HIF‐1α in AS, suggesting that HIF‐1α is the downstream factor of PC in the progression of AS (Figure 6O,P).

### 2.7 HIF‐1α Regulation by PC in Macrophages Played a Significant Role in Atherosclerosis

**Figure 7:**
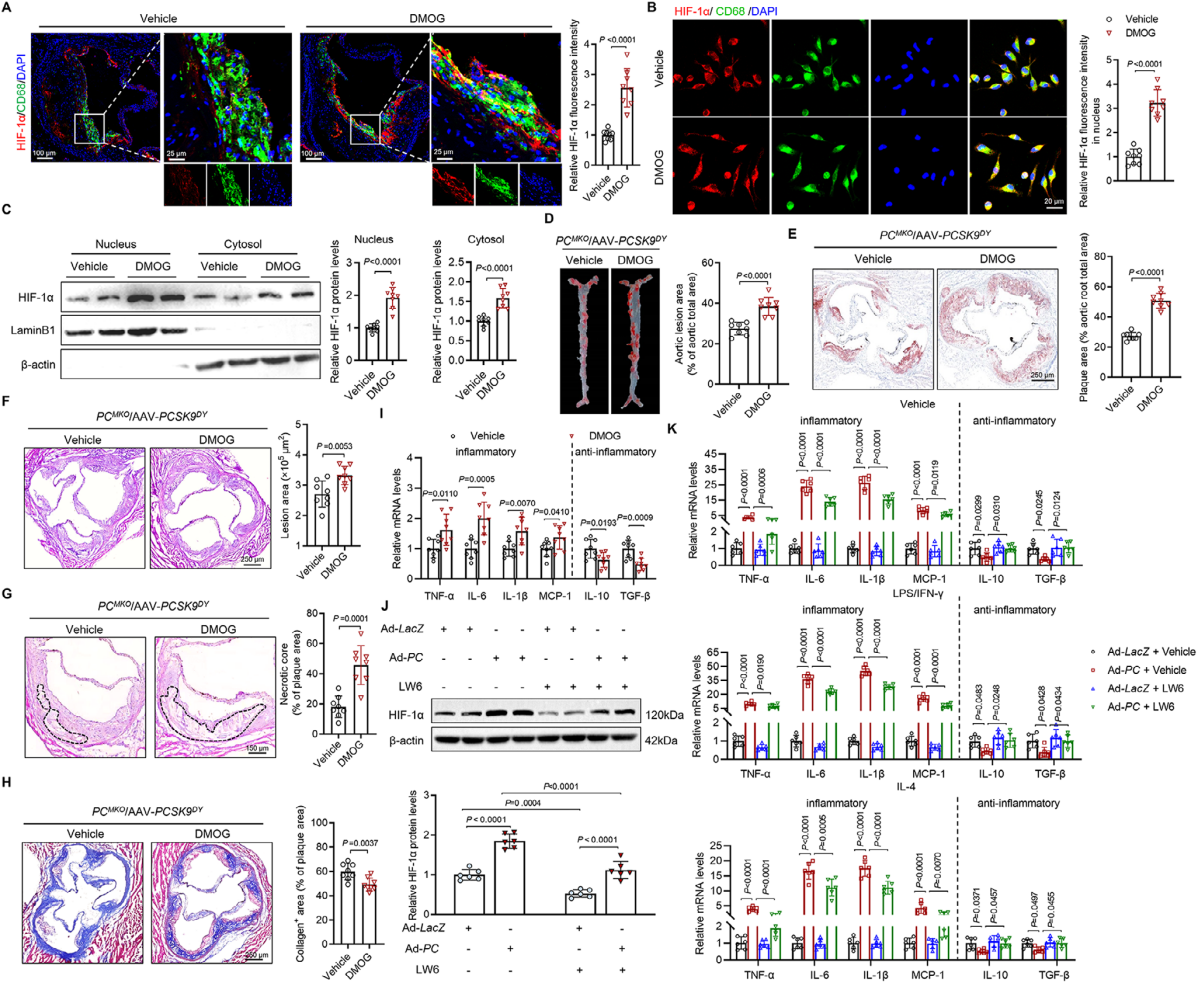
HIF‐1α regulation by PC in macrophages played a significant role in atherosclerosis. A–I) Male *PC^MKO^* mice were administrated with AAV‐*PCSK9^DY^*, followed by HFD feeding for 12 weeks and injected with HIF‐1α stabilizer DMOG (8 mg per mouse) or saline for the last 3 weeks (*n* = 8). BMDMs of *PC^MKO^* mice were collected for examination. A) Representative immunofluorescence images of HIF‐1α and CD68 in the plaques of aortic sinuses from *PC^MKO^* mice administrated with DMOG or saline. Fluorescence intensity of HIF‐1α in the nucleus of macrophages was quantified. Scale bars: 100 µm, enlarged: 25 µm. B) Representative immunofluorescence images of HIF‐1α and CD68 in BMDMs from *PC^MKO^* mice administrated with DMOG or saline. Fluorescence intensity of HIF‐1α in the nucleus of macrophages was quantified. Scale bars: 20 µm. C) Nuclear and cytosolic expression of HIF‐1α in BMDMs from *PC^MKO^* mice with DMOG or saline injection (*n* = 8). D) Representative images and quantification of the Oil Red O‐stained aortas from *PC^MKO^* mice. E) Representative images and quantification of the Oil Red O‐stained aortic root sections from *PC^MKO^* mice. Scale bars: 250 µm. F,G) Representative images of HE staining in aortic root sections from *PC^MKO^* mice. Lesion area F) and necrotic core area G) of the aortic root were quantified. Scale bars for (F): 250 µm; Scale bars for (G): 150 µm. H) Representative images and collagen quantification of the aortic roots stained with Masson staining. Scale bars: 250 µm. I) Quantification of selected mRNA in BMDMs from *PC^MKO^* mice administrated with DMOG or saline. J,K) Mouse primary BMDMs were transfected with Ad‐*LacZ* or Ad‐*PC* and treated with LW6 (20 µmol L^−1^) or DMSO for 24 h (*n* = 6). J) Representative HIF‐1α western blotting and protein quantification in BMDMs transfected with Ad‐*LacZ* or Ad‐*PC*, with or without LW6 treatment. K) Selected mRNA levels in BMDMs with different treatments, including LPS/IFN‐γ and IL‐4. Data are presented as means ± SD. A–I), Unpaired two‐tailed *t*‐test was used. J,K) Two‐way analysis of variance (ANOVA) with Tukey's correction was used. DMOG, dimethyloxalylglycine; AAV, adeno‐associated virus; HFD, high‐fat diet; LPS, lipopolysaccharides; IFN‐γ, interferon‐γ; IL‐4, interleukin‐4; oxLDL, oxidized low‐density lipoproteins; BMDM, bone marrow‐derived macrophage, HIF‐1, hypoxia‐inducible factor 1; PC, pyruvate carboxylase; HE, hematoxylin and eosin.

Given that our results identified HIF‐1α as the key effector of PC in macrophages, we investigated whether PC could exacerbate AS by upregulating HIF‐1α expression. Dimethyloxalylglycine (DMOG), a HIF‐1α stabilizer that inhibits hydroxylation from PHD and promotes HIF‐dependent transcription, was used in this study.^[^ ^22^ ^]^ We first determined the optimal concentration of DMOG (8 mg per mouse) and then administrated mice with DMOG every other day for 3 weeks following a 12‐week HFD and administration of AAV‐*PCSK9^DY^* (Figure S6A, S6B, Supporting Information). DMOG injection did not alter body weight or serum lipid levels of the mice (Figure S6C–S6G, Supporting Information). Additionally, DMOG increased HIF‐1α expression by suppressing PHD2 in macrophages and the aortic root of *PC^MKO^* and *PC^fl/fl^* mice, inducing nuclear translocation of HIF‐1α in macrophages (**Figure** **7**A–C; and Figure S6H, Supporting Information). Furthermore, DMOG reversed the mitigating effect of PC ablation on AS, resulting in larger plaque area in the aorta and aortic roots (Figure 7D,E; and Figure S6I–S6J, Supporting Information). Severe lesions and fragile structures were observed in the aortic roots of *PC^MKO^* and *PC^fl/fl^* mice following DMOG administration (Figure 7F–H; and Figure S6K–S6M, Supporting Information). In addition, the HIF‐1 pathway was activated by DMOG in *PC^MKO^* BMDMs, leading to an increased release of proinflammatory factors (Figure 7I; and Figure S6N, Supporting Information). However, DMOG merely induced AS in *PC^MKO^* and *PC^fl/fl^* mice which were fed with a chow diet and administrated with AAV‐*PCSK9^DY^* (Figure S7A–S7D, Supporting Information).

Next, we increased PC expression and administrated LW6 to inhibit HIF‐1α activity in primary macrophages. LW6 reduced the expression of HIF‐1α protein in macrophages, deactivating the HIF‐1 signaling pathway (Figure 7J; and Figure S8A, Supporting Information). The positive effect of PC on the HIF‐1 pathway to induce the inflammatory form of macrophages was reversed by LW6 (Figure 7K). Furthermore, LW6 inhibited foam cell formation and the binding and uptake of oxLDL in macrophages induced by PC overexpression (Figure S8B–S8D, Supporting Information). These findings suggest that macrophage PC promotes AS by promoting inflammatory responses through the HIF‐1 signaling pathway.

## 3 Discussion

**Figure 8:**
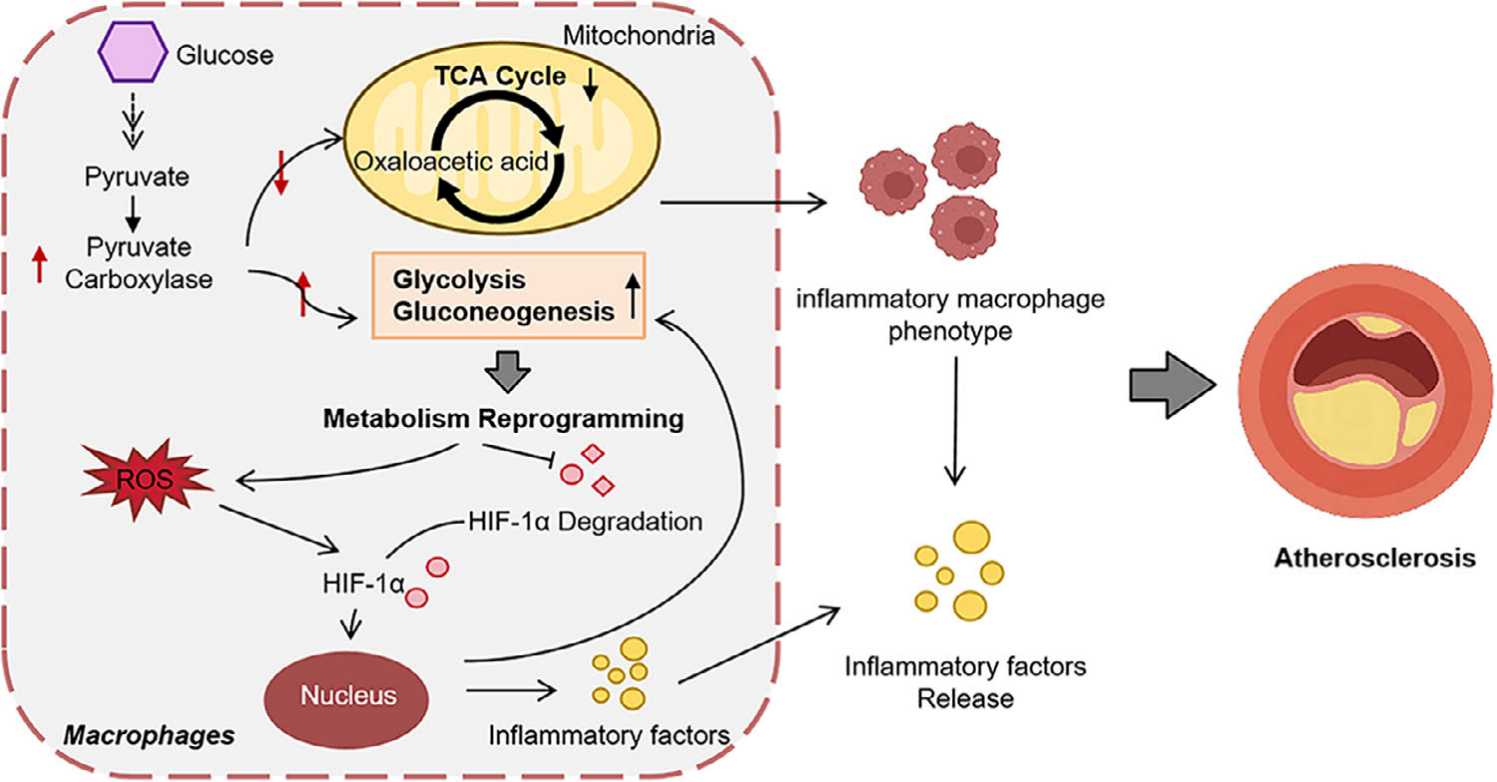
Pyruvate carboxylase in macrophages aggravates atherosclerosis by regulating metabolism reprogramming to promote inflammatory responses through HIF‐1 signal pathway. PC, pyruvate carboxylase; TCA cycle, tricarboxylic acid cycle; ROS, reactive oxygen species.

In this study, we constructed myeloid cell‐conditional PC knockout atherosclerotic mice and conducted in vivo and in vitro experiments to verify the role of PC in AS. PC is associated with plaque dynamics and the inflammatory macrophage transition during AS progression, reiterating its critical role in macrophage metabolism in atherogenesis. Our mechanistic results reveal that PC can initiate macrophage metabolic reprogramming, aggravating mitochondrial damage and ROS overproduction through HIF‐1 signaling pathway activation. Furthermore, inhibition of the PC and HIF‐1 pathways reduces the modulation of proinflammatory macrophage phenotype and ameliorates AS induction, providing novel insights for atherosclerotic treatment (**Figure** **8**).

AS is a chronic inflammatory disease, in which monocyte is recruited and differentiated into macrophages and foam cells, further releasing inflammatory cytokines and chemokines, causing the weakened fibrous cap and necrotic core formation.^[^ ^23^, ^24^ ^]^ Thus, the number and phenotype of macrophages influence the inflammatory state of the plaque, plaque stability, and ultimately, AS progression. Here, we reanalyzed the public RNA‐sequencing data from macrophages treated with VLDL‐sized emulsion particles and found that PC was upregulated, which was also observed in PBMCs from CAD patients and in HMDMs and BMDMs treated with oxLDL. These suggest PC participation in atherogenesis. Next, we constructed myeloid cell‐specific PC knockout mice and established two atherosclerotic mice models by administering AAV‐*PCSK9^DY^* and global knockout of *ApoE*. Interestingly, PC deletion ameliorated plaque formation and rupture in atherosclerotic models without altering the plasma lipid levels. Additionally, we observed reduced infiltration of inflammatory macrophages; however, the apoptosis and proliferation of macrophages were barely affected by PC deficiency. In BMDMs, PC enhanced the secretion of inflammatory factors such as TNF‐α, IL‐6, IL‐1β, and MCP‐1, while reducing the levels of anti‐inflammatory factors such as IL‐10 and TGF‐β. Furthermore, PC promoted the lipid uptake of macrophages and foam cell formation by affecting scavenger receptors. These data suggest that PC promotes the inflammatory macrophage phenotype rather than influencing macrophage numbers, leading to persistent chronic inflammation in AS.

PC is a tetrameric mitochondrial enzyme that catalyzes pyruvate carboxylation to oxaloacetate. In this study, we found that PC induced a different metabolic pattern in macrophages, characterized by stronger glycolytic capacity and reduced mitochondrial respiration. RNA sequencing results showed that glycolysis/gluconeogenesis, amino acid biosynthesis, and fatty acid and pyruvate metabolism were upregulated by PC in macrophages, suggesting that PC induced metabolic reprogramming in which the TCA cycle and OXPHOS were not the primary energy sources. In the TCA cycle, oxaloacetate is an essential intermediate that combines with acetyl coenzyme A to synthesize citrate to initiate energy production.^[^ ^25^ ^]^ The cycle requires replenishment to maintain its function because intermediates in the TCA cycle are transported out of the mitochondria for biosynthesis, a process known as anaplerosis.^[^ ^26^ ^]^ The carboxylation of pyruvate catalyzed by PC is an anaplerotic pathway that provides precursors for other biosynthetic processes while maintaining the integrity of the TCA cycle.^[^ ^14^ ^]^ However, as oxaloacetate can also be converted to phosphoenolpyruvate during gluconeogenesis, the anaplerotic pathway catalyzed by PC is essential for supporting gluconeogenesis.^[^ ^12^ ^]^ Accordingly, PC exerts ≈80% control over gluconeogenesis, indicating that increasing PC will more likely contribute to gluconeogenesis or TCA cycle replenishment rather than directly facilitating the TCA cycle for energy production.^[^ ^27^ ^]^ Notably, Cappel et al. found that PC loss impaired hepatic gluconeogenesis but promoted a more oxidized mitochondrial redox state without influencing mitochondrial number in hepatocytes.^[^ ^14^ ^]^ This implies an increased transfer of electrons from reductants to oxidants in the mitochondria, reflecting stronger activation of the ETC. Consistently, we found that PC ablation increased the maximal OCR and suppressed compensatory glycolysis in macrophages, indicating increased ETC capacity. This may result from a metabolic shift for energy production induced by PC under pathological conditions. Generally, inflammatory macrophages depend on anabolic metabolism to balance their energy requirements, characterized by high glycolytic rates and macromolecular synthesis, alongside a partially impaired TCA cycle.^[^ ^28^ ^]^ In contrast, anti‐inflammatory macrophages degrade nutrients via OXPHOS to efficiently generate ATP.^[^ ^29^ ^]^ Enhanced glycolysis and inhibition of the TCA cycle promote inflammatory responses, including inflammasome activation and the upregulation and release of proinflammatory factors by macrophages.^[^ ^30^ ^]^ In our study, an increased glycolysis with an altered metabolic form, upregulated glycolysis‐related genes and suppression of OXPHOS was triggered by PC, suggesting that PC facilitate metabolic reprogramming in macrophages which leads to the transition to an inflammatory macrophage phenotype, ROS overproduction and mitochondrial dysfunction. Excessive ROS generation and mitochondrial impairment are closely associated with AS.^[^ ^31^ ^]^ Furthermore, mitochondrial oxidative stress stimulates inflammatory macrophage phenotype, which activates inflammatory pathway to amplify AS.^[^ ^32^, ^33^ ^]^ Consistently, we observed that PC induced abnormal mitochondrial morphology, impaired membrane potential, and aggravated ROS accumulation in macrophages, exacerbating the suppression of respiration and thus initiating inflammatory responses. These findings provide further evidence that mitochondrial function plays a critical role in the AS development.

The HIF‐1 signaling pathway is vital for maintaining cellular homeostasis under hypoxia.^[^ ^34^ ^]^ As the pivotal regulator of hypoxia, HIF‐1 is a heterodimer protein consisting of HIF‐1α (active subunit) and HIF‐1β (unresponsive to hypoxia).^[^ ^35^ ^]^ Under hypoxia, the HIF‐1 protein becomes more active due to the prevention of hydroxylation by PHD and proteolytic degradation.^[^ ^36^ ^]^ PHD2 is the principal determinant of steady‐state HIF‐1α levels under normoxic conditions.^[^ ^37^ ^]^ Under physiological oxygen availability, PHD enzymes catalyze the C4‐hydroxylation of proline residues within the α‐subunits of HIF‐1 and HIF‐2.^[^ ^38^ ^]^ This post‐translational modification enhances HIF‐α binding affinity to the VHL protein, a key component of an E3 ubiquitin ligase complex, leading to HIF‐α ubiquitination and subsequent proteasomal degradation.^[^ ^20^ ^]^ Under hypoxic conditions, reduced PHD activity permits the stabilization of HIF‐α isoforms, which translocate to the nucleus, dimerize with HIF‐β, and drive the transcription of hypoxia‐responsive genes to mitigate hypoxic stress.^[^ ^39^ ^]^ In AS, HIF‐1α is upregulated in plaque regions infiltrated by macrophages and foam cells.^[^ ^40^ ^]^ Moreover, HIF‐1α deficiency can ameliorate AS and necrotic core formation by reducing macrophage necroptosis.^[^ ^41^ ^]^ In our study, the metabolism reprogramming initiated by PC activated the HIF‐1 pathway and increased HIF‐1α expression in macrophages. Notably, PC increased lactate accumulation in macrophages, which has been reported to suppress PHD2 activity via ROS overproduction and disrupt VHL function, thereby influencing HIF‐1α ubiquitination and proteasome degradation.^[^ ^42^, ^43^, ^44^, ^45^ ^]^ Furthermore, increased lactate levels from enhanced glycolysis can stimulate the accumulation of HIF‐1α in macrophages independently of hypoxia.^[^ ^46^ ^]^ Consistently, our findings suggest that increased glycolysis and lactate accumulation induced by PC impair PHD2 activity, reducing the ubiquitination and proteasome degradation of HIF‐1α. This allows HIF‐1α to translocate into the nucleus and activate the transcription of metabolism‐ and inflammation‐related genes in macrophages. DMOG is a competitive antagonist of α‐ketoglutarate that stabilizes HIF‐1α by inhibiting PHDs to prevent its hydroxylation.^[^ ^47^ ^]^ LW6 is an (aryloxyacetylamino) benzoic acid derivative that inhibits HIF‐1α accumulation and expression.^[^ ^48^, ^49^ ^]^ Herein, we observed that stabilizing HIF‐1α with DMOG aggravated atherosclerotic lesions in mice and reversed the protective effect of PC ablation on plaque progression. Furthermore, the inflammatory macrophage phenotype and over‐secretion of inflammatory factors triggered by PC were ameliorated by LW6, suggesting that HIF‐1α is a key effector of PC‐mediated AS exacerbation. Interestingly, we observed that certain glycolytic genes were also essential in the HIF‐1 pathway, suggesting that HIF‐1α can regulate glycolytic enzymes to promote glycolysis.^[^ ^50^ ^]^ Previous studies have demonstrated that inhibiting glycolysis with 2‐deoxyglucose (2‐DG) prevent activation of HIF‐1α and induction of IL‐1β in LPS‐treated macrophages, which emphasize the closed relation between glycolysis and HIF‐1 pathway in macrophages.^[^ ^51^ ^]^ Moreover, HIF‐1α deficiency in myeloid cells was found to reduce GLUT1 expression and ameliorate atherosclerosis in *Ldlr^−/−^* mice.HIF‐1α could upregulate PFKFB3 to promote glycolysis and initiate proinflammatory activation in macrophages.^[^ ^41^, ^52^ ^]^ Consistently, our data showed that PC activated glycolysis‐ and HIF‐1‐related genes, suggesting that HIF‐1α upregulation by PC through aerobic glycolysis promotes a glycolysis‐dominant metabolic shift, establishing a feedback loop between HIF‐1α expression and glycolysis. However, further investigations are required to validate this hypothesis.

Our study suffers from several limitations. First, although the *Lyz2‐Cre* strain effectively targets macrophages, its specificity is limited as it potentially affects neutrophils and dendritic cells. Future studies may consider using F4/80‐cre for greater specificity. Second, while our study identifies that the HIF‐1 pathway is initiated by metabolic reprogramming mediated by PC, further experiments using CRISPR interference technology to specifically target key genes in the HIF‐1 signaling pathway and observing the impact on atherosclerotic lesions are needed. Moreover, multiomics analysis should be considered to apply in future studies to demonstrate the metabolic changes induced by PC in macrophages during the progression of AS. Finally, our findings underscore the therapeutic potential of HIF‐1α inhibition. Recent studies also have explored the effect of drugs or natural bioactive substances directly targeting PC on several inflammatory diseases.^[^ ^53^ ^]^ However, the optimal dosing regimen and long‐term effects in clinical practice require further investigation.

In conclusion, our study demonstrates that PC is pivotal in the progression of AS by modulating macrophage metabolism and inflammatory responses. Increased PC expression in macrophages promoted a transition to an inflammatory macrophage phenotype and a metabolic shift toward glycolysis, thereby aggravating mitochondrial dysfunction and ROS overproduction in AS. Mechanistically, PC reduced HIF‐1α ubiquitination and protected HIF‐1α from proteasome degradation, ultimately activating the HIF‐1 signaling pathway. Inhibition of PC and HIF‐1α mitigated macrophage inflammatory responses, reducing plaque instability and atherogenesis. These findings underscore the critical role of macrophage metabolism in AS and suggest the therapeutic potential of PC for the treatment of AS.

## 4 Experimental Section

### Human Samples

Patients with CAD and healthy individuals were recruited from Department of Cardiovascular Surgery, Nanfang Hospital, from January 2023 to March 2025. CAD patients were diagnosed according to the Guideline for the Management of Patients With Chronic Coronary Disease of the American College of Cardiology/American Heart Association (ACC/AHA). Exclusion criteria were as follows: a history of stroke, carcinoma, diabetes mellitus, infection, autoimmune diseases, hematological disorders, or severe renal insufficiency. Healthy individuals who underwent routine physical examinations in Nanfang Hospital and admitted no history of cardiovascular diseases or diabetes were recruited. Human blood samples were collected from all enrolled participants. PBMCs were isolated and purified using the Monocyte Isolation Kit II (Miltenyi Biotec, San Diego, CA). The baseline characteristics of subjects were presented in Table S1 (Supporting Information). All patients were informed and agreed to participate with formal consent obtained according to the Declaration of Helsinki. The Ethics Committee of Nanfang Hospital, Southern Medical University approved this investigation (NFEC‐2023‐488).

### Animal Studies

All animal experiments were approved by the Animal Research policies of the Southern Medical University Committee in Nanfang Hospital (approval number NFYY‐2022‐0143) and followed the Guide for the Care and Use of Laboratory Animals of the National Institute of Health in China.

Mice expressing the PC gene flanked by loxp sites (*PC^fl/fl^*, stock no. T008093) were generated by GemPharmatech Co., Ltd. (Jiangsu, China). *Lyz2*‐iCre mice [*Lyz2^em1Cin(iCre)^*; stock no. T003822] and *ApoE^−/−^* (stock no. T001458) mice were purchased from GemPharmatech Co., Ltd. Macrophage‐specific PC knockout mice, the *Lyz2*‐Cre/*PC^fl/fl^* (*PC^MKO^*) mice, were generated by crossbreeding *PC^fl/fl^* mice with *Lyz2*‐Cre mice. All mice were bred on a C57BL/6J background before experiments. The subsequent generations of mice were genotyped by PCR with primers listed in Table S2 (Supporting Information).

Eight‐week‐old male mice were fed a chow diet of high‐fat diet (HFD, 12.5% sucrose, 45% fat, and 2% cholesterol) for 12 weeks. For DMOG administration, male mice were randomized to DMOG (2/4/6/8/10 mg per mouse, MedChemExpress) or vehicle and accepted intraperitoneal administration every other day for 3 weeks to determine the optimal dose. Consequently, a dose of 8 mg per mouse was selected for the experiments. Mice received intraperitoneal DMOG administration every other day for 3 weeks following a 12‐week HFD and administration of AAV‐*PCSK9^DY^*. All mice were raised in the SPF animal laboratory at a room temperature of 21–24 °C and a 12‐h light/dark cycle.

### Bone Marrow Transplantation

Six‐week‐old male C57BL/6J *ApoE^−/−^* mice were lethally irradiated with a total radiation dose of 1100 Rads (two doses of 550 Rads, 4‐h apart) by a standard irradiator (Faxitron MultiRad225). The bone marrow cells extracted from the tibiae and femurs of male *PC^fl/fl^* or *PC^MKO^* mice were administrated into the tail vein of the irradiated recipient mice (5 × 10^6^ cells per mouse). The mice were maintained on antibiotic‐containing water (sulfatrim, 4 µg mL^−1^) and a chow diet for 4 weeks. Peripheral blood was collected for the PCR analysis of hematologic chimerism using the primers listed in Table S2 (Supporting Information). After the confirmation of successful transplantation, the mice were fed a HFD for 12 weeks.

### Adeno‐Associated Virus (AAV) Construction and Transfection

The pAAV/D374Y‐*hPCSK9* (*PCSK9^DY^*) plasmid driven by the ApoEHCR‐hAAT promoter was generously provided by Dr. Bentzon (Addgene plasmid #58 379). For the production of AAV8 adenoviruses (AAV‐*PCSK9^DY^*), the plasmids were separately co‐transfected with the pAAV2/8 trans‐plasmid containing the AAV rep and cap genes and the pAAV helper plasmid into HEK293T cells. AAV‐*LacZ* was applied as a negative control. Viral titres were evaluated by PCR using vector‐specific primers. AAV‐*PCSK9^DY^* (2 × 10^11^ vector genomes per mouse) was administrated into mice by a single tail‐vein injection. Simultaneously, the mice were fed a chow diet or HFD for 12 weeks since the AAV injection.

### Cell Culture, Treatment, and Transfection

HMDMs were isolated as previously described.^[^ ^17^ ^]^ Human PBMCs were obtained from the blood samples of healthy volunteer donors using Percoll density‐gradient centrifugation, which then were differentiated into HMDMs in RPMI‐1640 medium supplemented with 10% fetal calf serum (FCS), 1% HEPES, 1% penicillin/streptomycin, and 100 ng mL^−1^ of macrophage colony‐stimulating factor (M‐CSF) (Invitrogen, Carlsbad, CA) for 6 days.

Mouse BMDMs were isolated from aged 8–12‐week‐old male mice, whose tibiae and femurs were collected to extract bone marrow cells. The isolated bone marrow cells were cultured for 7 days in Dulbecco's Modified Eagle Medium (DMEM) with 10% fetal bovine serum (FBS), 1% HEPES, 1% penicillin/streptomycin, and 30% L929 conditioned medium, which serves as a source of M‐CSF to promote differentiation into BMDMs. To induce a proinflammatory macrophage phenotype, BMDMs were treated with 100 ng mL^−1^ LPS (L4516, Sigma‐Aldrich) and 100 ng mL^−1^ IFN‐γ (I4777, Sigma‐Aldrich) in serum‐free medium. To induce an anti‐inflammatory macrophage phenotype, BMDMs were treated with 5 ng mL^−1^ IL‐4 (SRP3211, Sigma‐Aldrich). To examine the influence of lactate on macrophages, BMDMs were stimulated by 5 mm lactate (L6402, Sigma‐Aldrich) for 12 h.

MASMCs and MAECs were obtained as previously described.^[^ ^54^ ^]^ MASMCs were isolated from the aorta of 10‐week‐old male mice via collagenase digestion and cultured in DMEM with 10% FBS and 1% penicillin/streptomycin. MASMCs from generations 4–8 were used for examinations. For MAECs isolation, mouse aorta was washed with cold PBS, then perfused with 1 mL of QIAzol Lysis reagent (79 306, Qiagen) within 1 min. After washing with PBS, the cells were centrifuged and collected, consisting primarily of MAECs. The successful isolation of MASMCs and MAECs was confirmed using quantitative reverse transcription polymerase chain reaction (RT‐qPCR) to assess the expression of cell markers. All cells were maintained at 37 °C in a humidified incubator with a gas ratio of 95% O_2_ and 5% CO_2_.

To examine the post‐transcriptional processes of HIF‐1α protein, protein synthesis inhibition, cycloheximide (CHX, 50 µg mL^−1^, MedChemExpress), together with lysosomal inhibitor chloroquine (CQ, 20 µmol L^−1^, MedChemExpress), or proteasomal inhibitor MG132 (10 µmol L^−1^, MedChemExpress). For HIF‐1α inhibition, BMDMs were treated with 20 µmol L^−1^ LW6 (MedChemExpress). To overexpress HIF‐1α, primary BMDMs were transfected with HA‐HIF‐1α plasmids (Shenzhen DINGKE Biotechnology Co., LTD) using Lipofectamine 2000 reagent (Invitrogen) based on the manufacturer's instructions.

### Adenovirus Construction and Infection

PC adenovirus was generated using the AdEasy Vector System Kit (Agilent Technologies) according to the manufacturer's instructions. The coding sequence of murine PC was subcloned into the pCMV vector using the following primers: forward, 5′‐GCCCAGAAGTTGCTACATTACCT‐3′ and reverse, 5′‐CTCACATTGACAGGGATTGGA‐3′. Recombinant adenoviruses were purified and titrated to yield a viral count of 1 ×10^11^ pfu mL^−1^. Each adenovirus was then used for infecting the cells in culture without serum or antibiotics for 6 h, after which the cells were transferred to fresh full medium and cultured for another 18 h. Protein and RNA levels were examined to verify PC overexpression. LacZ‐harboring adenovirus (Ad‐*LacZ*) was used as a negative control.

### Serum Analysis

After an overnight fast, blood samples were harvested from anesthetized mice (pentobarbital sodium, i.p.). Total serum cholesterol, triglyceride, low‐density lipoprotein (LDL), or high‐density lipoprotein (HDL) levels were assessed using a fully automated clinical chemistry analyzer (Chemray800, Rayto Life and Analytical Sciences Co., Ltd., Shenzhen, China). The serum concentrations of TNF‐α, IL‐6, IL‐1β, IL‐10, TGF‐β were assayed using the ELISA kits procured from R&D Systems (Minneapolis, MN).

### Histological Examinations

After sacrifice, mice were perfused with saline through the left ventricle to prevent blood clotting. The aorta was dissected up to the left ventricle and photographed immediately. Then, the aorta was either incised longitudinally for oil red O staining or fixed in 4% paraformaldehyde, embedded in OCT or paraffin and sectioned. Then aorta sections were examined by Hematoxylin and Eosin (H&E), Masson stains according to the manufacturer's instructions.

Oil red O staining: The whole mouse aorta opened longitudinally from bottom to top and the aortic root slice was examined by oil red O staining. The aortic tissues and slices were soaked in 60% isopropanol for 2 min, stained with oil red O for 5–10 min and toned with 60% isopropanol. The slices still needed counterstaining with hematoxylin, differentiation, and mounting with glycerin gelatin. Images of atherosclerotic plaques in the whole aorta and the aortic root were captured by an Olympus upright microscope (Japan).

HE staining: The paraffin‐embedded sections were deparaffinized and dehydrated, subsequently stained hematoxylin for 5 min, rinsed with water and differentiated. After the slices turned blue, alcoholic eosin staining was performed. Images of aortic sections were captured by an Olympus upright microscope (Japan).

Masson staining: The aorta sections were deparaffinized with xylene and ethanol until fully dehydrated. The sections were stained by Weigert's iron hematoxylin, Ponceau acid fuchsin solution, 1% phosphomolybdic acid aqueous solution, and blue aniline solution in sequence. Then the sections were sealed and observed by an Olympus upright microscope (Japan).

### Immunofluorescence

OCT‐embedded aortic sections and paraformaldehyde‐fixed cells were permeabilized with 0.1% triton X‐100, blocked with 5% BSA at room temperature and then incubated with primary antibodies at 4 °C overnight. After washed with PBS, the sections or cells were incubated with secondary antibodies. Nuclei were counterstained with 4′,6‐diamino‐2‐phenylindole (DAPI). The antibodies used in the study were as follows: anti‐CD68 (1:100, BA3638, Boster Biological Technology), anti‐PC (1:200, ab110314, Abcam), anti‐HIF‐1α (1:50, sc‐13515, Santa Cruz Biotechnology), anti‐Ki67 (1:50, sc‐15402, Santa Cruz Biotechnology). Immunostaining images of cells and tissues were acquired using a fluorescence confocal microscope (LSM 980, Carl Zeiss, Oberkochen, Germany).

### Binding and Uptake of oxLDL

In brief, oxLDL was labeled with l,l’‐dioctadecyl‐3,3,3′,3′‐tetramethyl‐indocarbocyanine perchlorate (Dil). The cells were incubated with Dil‐oxLDL (10 µg mL^−1^) for 2 h at 4 °C or 4 h at 37 °C, which were then fixed with 4% paraformaldehyde for 10 min and subsequently stained with DAPI. After several washes with PBS, the cells were imaged using a confocal microscope (Zeiss LSM980), and the fluorescence intensity was quantified using ImageJ software.

### Terminaldeoxy Nucleotidyl Transferase‐Mediated dUTP‐Biotin Nick End Labeling (TUNEL) Analysis

A TUNEL Apoptosis Assay Kit (C1089, Beyotime Biotechnology) was used to detect the apoptotic level in aortic roots. Frozen sections of aortic roots were immersed with 0.1% Triton X‐100, then incubated with the TUNEL reagent for 1 h at 37 °C. The nuclei were stained with DAPI. Apoptotic cells were observed by a confocal microscope (Zeiss LSM980) and quantified by the ImageJ software.

### In Situ Dihydroethidium (DHE) Staining

In situ DHE staining (R353922, Aladdin) was performed to evaluate superoxide production. BMDMs were incubated with 100 µm DHE staining solution at room temperature in darkness for 90 min. Images were captured under a confocal microscope (Zeiss LSM980).

### Seahorse Assays

OCR and ECAR were measured using a Seahorse XF Cell Mito Stress Test Kit (No. 103015‐100, Agilent Technologies) and a Seahorse XF Glycolytic Rate Assay Kit (No. 103344‐100, Agilent Technologies) with an Agilent Seahorse XFe96 Analyzer according to the manufacturer's instructions. In brief, BMDMs were seeded at a density of 2×10^4^ cells per well in XFe96 microplates. After 16 h, the growth medium were changed to XF assay medium (XF DMEM medium with 1 mmol L^−1^ pyruvate, 2 mmol L^−1^ glutamine, and 10 mmol L^−1^ glucose) and then BMDMs were incubated in a 37 °C CO_2_‐free incubator for 1 h. For cell mito stress test, oligomycin (1.5 µmol L^−1^), carbonylcyanide‐4‐(trifluoromethoxy) phenylhydrazone (FCCP; 1.0 µmol L^−1^), and a mixture of rotenone and antimycin A (Rot+Ant; 0.5 µmol L^−1^) were added into the plate at the indicated time according to the protocol. For glycolytic rate assay, Rot+Ant (0.5 µmol L^−1^) and 2‐deoxy‐D‐glucose (2‐DG; 50 mmol L^−1^) was added into each well according to the protocol. Finally, OCR and ECAR were analyzed with protein content normalized on the Seahorse XFe96 Analyzer according to the manufacturer's instructions.

### Lactate Level Measurement

Lactate levels were quantified using the L‐Lactate Assay Kit (ab65330, Abcam) according to the manufacturer's instructions. The culture medium was diluted and then mixed with L‐lactate assay reagent in a 96‐well plate. The plate was incubated at room temperature for 30 min. The absorbance at 570 nm wavelength was measured with the standard microplate reader.

### TEM

Mitochondrial morphology was observed by TEM. Small BMDMs fragments (1 mm^3^ in volume) were fixed in 2.5% glutaraldehyde at 4 °C for 2–4 h, followed by three 15‐min rinses in 0.1 m phosphate buffer (pH 7.4). The fragments were then subjected to postfixation in 1% osmium tetroxide in 0.1 m phosphate buffer (pH 7.4) at 20 °C for 2 h. Following another rinse in phosphate buffer, the fragments were dehydrated through a graded ethanol series (50%, 70%, 90%, and 100%). Permeabilization was carried out using a 1:1 mixture of propylene oxide and Epon 812 for 2–4 h, followed by an overnight treatment with a 1:2 mixture of propylene oxide and Epon 812. After polymerization at 37 °C, the fragments were sectioned into ultrathin slices (80 nm) using an ultramicrotome (UC7, Leica, Germany). The sections were then stained with 2% uranyl acetate and lead citrate and examined under an electron microscope (JEM‐1400, Japan).

### Measurements of Mitochondrial Membrane Potential

To assess alterations in mitochondrial membrane potential, BMDMs were stained with a JC‐1 Mitochondrial Membrane Potential Assay Kit (C2006, Beyotime) according to the manufacturer's instructions. The nuclei were counterstained with DAPI. Cells were imaged under a confocal microscope (Zeiss LSM980).

### Intracellular Lipid Deposition

Oil Red O staining was used to evaluate intracellular fat content. In brief, macrophages were fixed with 4% paraformaldehyde at room temperature, which then were stained with 0.3% oil Red O solution (dissolved in isopropanol:water, 3:2) for 30 minutes. After rinsing with isopropanol and PBS, cells were observed and imaged under a light microscope (IX73). Analysis of oil Red O positive area was performed using the ImageJ software. The levels of free cholesterol and cholesteryl esters were measured with the Cholesterol and Cholesteryl Ester Colorimetric Assay Kit II (Biovision, Mountain View, CA).

### RNA Sequencing

Total RNA was extracted from BMDMs by the TRIzol reagent (Invitrogen Life Technologies) according to the manufacturer's instructions. RNA integrity was evaluated using the RNA Nano 6000 Assay Kit of the Agilent Bioanalyzer 2100 system (Agilent Technologies). Library preparation was performed by Novogene (Beijing, China). RNA sequencing was performed following the manufacturer's instructions, with 1 µg of RNA as input material for library preparation by using the NEBNext UltraTM RNA Library Prep Kit for Illumina (New England Biolabs). The quality control was assessed with the Agilent Bioanalyzer 2100 system. DNA fragments were aligned using 150‐bp paired‐end sequencing on an Illumina HiSeq 2500 platform. Differential expression analysis was performed with the DESeq2 R package. Genes with an adjusted *p*‐value < 0.05 and |fold‐change| ≥ 2 were considered differentially expressed genes. Multiple test correction was performed using the Benjamini–Hochberg method. Kyoto Encyclopedia of Genes and Genomes (KEGG) and Gene Ontology (GO) enrichment analysis of differentially expressed genes was conducted with the clusterProfiler R package, which corrects gene length bias.

### Reverse Transcription Quantitative Polymerase Chain Reaction (RT‐qPCR)

Total RNA was extracted from cells by TRIzol (Ambion, USA). RNA reverse transcription to generate cDNA was conducted with the reverse transcription reagent Evo M‐MLV RT Master Mix (Agbio, Hunan, China). Gene expression was evaluated by a LightCycler 480 real‐time PCR instrument (Roche, Indianapolis, IN). The reaction conditions were set using a fluorescent quantitative PCR kit (SYBR Green Premix, Agbio, Hunan, China). The results were analyzed with the 2^−△△Ct^ method and normalized to β‐actin gene expression. The primers are listed in Table S3 (Supporting Information).

### Protein Extraction and Western Blotting

Proteins were extracted from cells and tissues using RIPA lysis buffer with a protease inhibitor (Merck, Darmstadt, Germany). Specifically, liver tissues were homogenized using a sample freezing grinder (LUKYM‐I, Lu ka Co., Ltd., Guangzhou, China) at a frequency of 75 Hz, with 30 s of grinding followed by a 20‐s pause, completing the process in 4 cycles. Nuclear and cytoplasmic protein were isolated using the PARIS Kit (AM1921) according to the manufacturer's instructions (Ambion, Carlsbad, CA). After quantified by BCA assay (Thermo Fisher Scientific, Waltham, MA), proteins were resolved in 6%–12% SDS‐PAGE gels and then transferred onto polyvinylidene fluoride membranes (Millipore, USA). The membranes were blocked with 5% nonfat milk for 1 h at room temperature and then incubated with primary antibodies overnight at 4 °C. The primary antibodies used were as follows: PC (1:5000, 16588‐1‐AP, Proteintech), HIF‐1α (1:1000, PB9253, Boster Biological Technology), Ubiquitin (1:1000, 10201‐2‐AP, Proteintech), Lamin B1 (1:1000, #13 435, Cell Signaling Technology), LDL‐R (1:500, sc‐11824, Santa Cruz Biotechnology), CD36 (1:1000, 18836‐1‐AP, Proteintech), SR‐A1 (1:2000, 24655‐1‐AP, Proteintech), ABCG1 (1:1000, ab52617, abcam), PHD2 (1:5000, 19886‐1‐AP, Proteintech), and β‐actin (1:20 000, 66009‐1‐Ig, Proteintech). After washing with TBST, the membranes were incubated with corresponding secondary antibodies. Signals were detected by the standard ECL kit (Thermo Fisher Scientific), and densitometry was evaluated with ImageJ_v1.8.0 software.

### Ubiquitination Analysis

Cells were lysed in IP buffer containing 1% SDS and denatured at 95 °C for 10 min. The lysates were diluted with IP buffer to a 0.1% SDS concentration. After centrifugation for 10 min at 12 000 × g at 4 °C, the supernatants were immunoprecipitated with protein A/G magnetic beads and the corresponding antibodies overnight at 4 °C. Immunoprecipitates were eluted by boiling in 2× SDS loading buffer and then analyzed by western blotting.

### PC Enzymatic Activity Assay

BMDMs were isolated from wild type mice and stimulated with oxLDL, or isolated from *ApoE^−/−^* mice fed with a chow diet or HFD. Cells were lysed using the buffer in the PC Activity Assay kit (CAK1262, Cohesion, UK) according to the manufacturer's instructions. PC enzymatic activity was measured using colorimetric assays.

### Statistical Analysis

Quantitative data were expressed as the mean ± standard deviation (SD). Statistical analyses were performed using R (version 4.3.1) or GraphPad Prism (version 9.0; GraphPad software, CA). All data were checked for normality using the Shapiro–Wilk test or Kolmogorov–Smirnov test. A two‐tailed unpaired Student's *t*‐test and Mann–Whitney *U* test with exact method were used for comparison between two groups. Comparisons among three or more groups were assessed by one‐way analysis of variance (ANOVA) or two‐way ANOVA and confirmed by Tukey's multiple comparisons test or Bonferroni's multiple comparisons test. A *p*‐value <0.05 was considered as statistically significant.

## Conflict of Interest

The authors declare no conflict of interest.

## Author Contributions

L.N.Z., R.L.W., R.X.L., and M.R.Z. contributed equally to this work. S.Y.Z., X.L., and F.Z.H. conceived the project and designed the experiments. F.Z.H., L.N.Z., R.L.W., R.X.L., and M.R.Z. performed main experiments and data collection. F.Z.H., L.N.Z., R.L.W., R.X.L., M.R.Z. L.Z., B.F.L., J.L.L., and D.S.L. participated in data analysis and interpretation. X.X.H., T.X.L., Q.B.P, S.P.H., and J.L. collected clinical samples and partially performed essential experiments. K.L., T.X.L., Y.Y.L., S.P.H., and J.L. helped to design the study and provided constructive comments. F.Z.H., L.N.Z., R.L.W., R.X.L., and M.R.Z. drafted the manuscript. S.Y.Z., X.L., and F.Z.H. revised the manuscript and oversaw the experiments. All authors discussed the results and commented on the manuscript.

## Supporting information



Supporting Information

Supporting Information

## Data Availability

The data that support the findings of this study are available from the corresponding author upon reasonable request.
