# Supplementary material for: Pyruvate Carboxylase in Macrophages Aggravates Atherosclerosis by Regulating Metabolism Reprogramming to Promote Inflammatory Responses Through the Hypoxia‐Inducible Factor‐1 Signaling Pathway
Source: Adv Sci (Weinh). 2025 May 20;12(29):e17128. doi: 10.1002/advs.202417128 (PMC12362773; doi:10.1002/advs.202417128)
Supplement: Supplementary file 1 — Supporting Information [file ADVS-12-e17128-s001.docx]

Supporting Information

Title: Pyruvate Carboxylase in Macrophages Aggravates Atherosclerosis by Regulating Metabolism Reprogramming to Promote Inflammatory Responses through the Hypoxia-Inducible Factor-1 Signaling Pathway

Ling-Na Zhao, Rui-Ling Wang, Ran-Xin Liu, Meng-Ru Zheng, Li Zhao, Bao-Feng Li, Jia-Le Li, De-Shen Liu, Xiao-Xia He, Qin-Bao Peng, Kai Li, Tian-Xiao Lin, Ying-Ying Liu, Sheng-Ping He, Jun Lu, Shao-Yi Zheng,* Xiu Liu,* and Fang-Ze Huang*

((Please insert your Supporting Information text/figures here. Please note: Supporting Display items, should be referred to as Figure S1, Equation S2, etc., in the main text…)

**Supplementary Tables**

**Table S1. Baseline characteristics of the subjects in this study**

|  | Non-CAD | CAD |
| --- | --- | --- |
| All (n) | 27 | 53 |
| Male (n, %) | 15 (55.6) | 35 (66.0) |
| Age (years) | 49.1±11.6 | 63.1±4.6 |
| BMI (kg/m^2^) | 21.8±2.4 | 23.1±2.7 |
| Total cholesterol (mmol/L) | 3.87±1.71 | 4.51±2.18 |
| Triglycerides (mmol/L) | 1.39±0.62 | 1.78±0.80 |

Data are presented as the mean ± SD. CAD, coronary artery diseases; BMI, body mass index

**Table S2. Primers used for genotyping in this study**

| Gene | Forward Primers (5′-3′) | Reverse Primers (5′-3′) |
| --- | --- | --- |
| *PC^fl/fl^* F1 | TGCAGCTTCCTATAAGATTTGTACCAG | CCCTGATACTGAGCAAGCTAATCTTGG |
| *PC^fl/fl^* F2 | GCCCATCAGTCCTCAACTCAATG | GGGTGAGAACAGTAATCACCAAGGC |
| *Lyz2-iCre* | AGTGCTGAAGTCCATAGATCGG | CTGATTCTCCTCATCACCAGG |
| *ApoE^-/^*^-^ | TGCCTAGTCTCGGCTCTGAACTAC | CAACCTGGGCTACACACTAATTGAG |
| *ApoE* chimaera F1R1 | GCCCATCAGTCCTCAACTCAATG | GGGTGAGAACAGTAATCACCAAGGC |
| *ApoE* chimaera F1R2 | GCCCATCAGTCCTCAACTCAATG | CCCTGATACTGAGCAAGCTAATCTTGG |

**Table S3. Primers used for RT-PCR in this study**

| Gene | Forward Primers (5′-3′) | Reverse Primers (5′-3′) |
| --- | --- | --- |
| PC (human) | CGACGGCGAGGAGATAGTGT | GCTTCATCTGCTTTCTGCCG |
| PC (mouse) | GCCCAGAAGTTGCTACATTACCT | CTCACATTGACAGGGATTGGA |
| TNF-α (mouse) | CCTCTCATGCACCACCATCA | GCATTGCACCTCAGGGAAGA |
| IL-6 (mouse) | GAGACTGGGGATGTCTGTAGC | TCACCAGCATCAGTCCCAAG |
| IL-1β (mouse) | CTTTCCCGTGGACCTTCCAG | AATGGGAACGTCACACACCA |
| MCP-1 (mouse) | TAAAAACCTGGATCGGAACCAAA | GCATTAGCTTCAGATTTACGGGT |
| IL-10 (mouse) | GCATGGCCCAGAAATCAAGG | AATCGATGACAGCGCCTCAG |
| TGF-β (mouse) | TGATACGCCTGAGTGGCTGTCT | CACAAGAGCAGTGAGCGCTGAA |
| Pfk1 (mouse) | CAGATCAGTGCCAACATAACCAA | CGGGATGCAGAGCTCATCA |
| Pkm (mouse) | GCTGTTTGAAGAGCTTGTGC | TTATAAGAGGCCTCCACGCT |
| Eno1 (mouse) | GCCTCCTGCTCAAAGTCAAC | CATGGAGGGCGGATTGGAA |
| Ldha (mouse) | CAAAGACTACTGTGTAACTGCGA | TGGACTGTACTTGACAATGTTGG |
| Tpi1 (mouse) | CCTTCCATTGGTTTGGGCTG | AATACAGGGGCTTTGGCACC |
| Aldoa (mouse) | AGTCCACCGGAAGCATTGC | CAGCCCCTGGGTAGTTGTC |
| Eno1b (mouse) | CCTACTGCCAGAAATTCGCC | GAGACACCCTTCCCCATGAA |
| Hk2 (mouse) | ATGATCGCCTGCTTATTCACG | CGCCTAGAAATCTCCAGAAGGG |
| HIF-1α (human) | GAACGTCGAAAAGAAAAGTCTCG | CCTTATCAAGATGCGAACTCACA |
| HIF-1α (mouse) | GGTTCCAGCAGACCCAGTTA | AGGCTCCTTGGATGAGCTTT |
| β-actin (human) | atgggtcagaaggattcctat | aagagtgcctcagggcag |
| β-actin (mouse) | GTTGGAGCAAACATCCCCCA | CGCGACCATCCTCCTCTTAG |
| Pdha1 (mouse) | GAAATGTGACCTTCATCGGCT | TGATCCGCCTTTAGCTCCATC |
| Aco2 (mouse) | ATCGAGCGGGGAAAGACATAC | TGATGGTACAGCCACCTTAGG |
| Idh1 (mouse) | ATGCAAGGAGATGAAATGACACG | GCATCACGATTCTCTATGCCTAA |
| Ogdh (mouse) | AGGGCATATCAGATACGAGGG | CTGTGGATGAGATAATGTCAGCG |
| Sdhd (mouse) | TGGTCAGACCCGCTTATGTG | GGTCCAGTGGAGAGATGCAG |
| Fh1 (mouse) | GAATGGCAAGCCAAAATTCCTT | TCTTACGGTCTGAGCACCATAA |
| Ndufa7 (mouse) | TCCGCTACTCGCGTTATCCA | GATTGAGGGAGGCACAACTTC |
| Ndufb9 (mouse) | GGTACTTTGCTTGCTTGATGAGA | TGGGAAGATATACGGCTGAGG |
| Ndufa3 (mouse) | ATGGCCGGGAGAATCTCTG | AGGGGCTAATCATGGGCATAAT |
| Cox6b1 (mouse) | ACTACCTGGACTTCCACCG | ACCCATGACACGGGACAGA |
| Cox7c (mouse) | ATGTTGGGCCAGAGTATCCG | ACCCAGATCCAAAGTACACGG |
| Uqcr1 (mouse) | TCTGCACATGCGTAGTGCTC | CCGTTGATGTAAGGCACCCA |

**Supplemental Figure and Figure Legends**

**
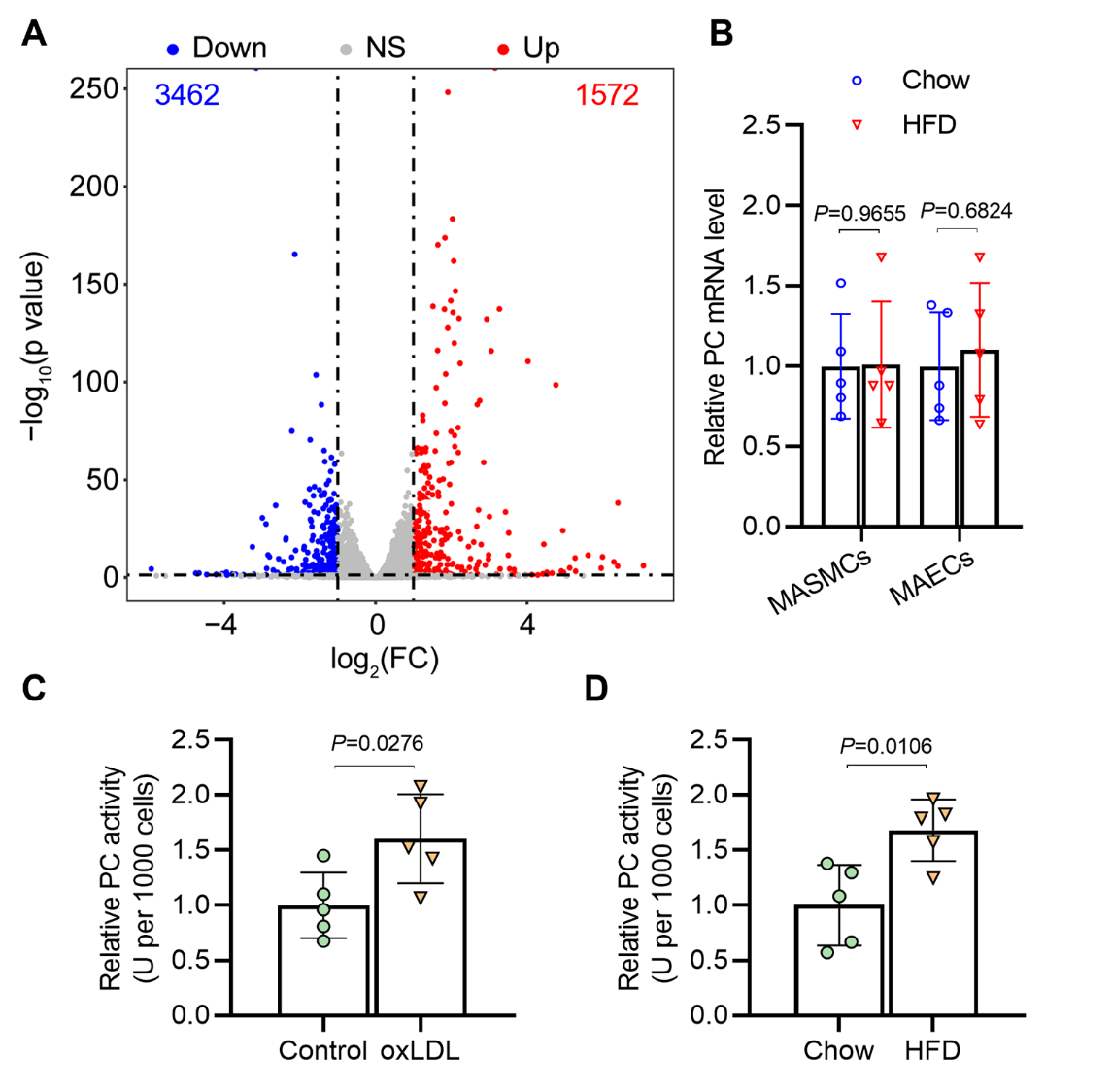
**

**Figure S1. PC was upregulated in macrophages of atherosclerotic mice. (A)** Volcano plots of RNA-sequencing (GSE203250) reanalysis in RAW 264.7 macrophages treated with VLDL-sized emulsion particles compared to that treated with vehicle (n=3). Differentially expressed genes were defined as those with a greater than 2-fold change and an *P* value of less than 0.05. Upregulated and downregulated differentially expressed genes were colored in red or blue, respectively. **(B)** PC mRNA levels in MASMCs and MAECs isolated from male *ApoE^-/-^* mice fed with a chow diet or HFD (n=5). **(C)** PC activity in mouse primary BMDMs treated with oxidized low-density lipoproteins (oxLDL, 80 μg mL^-1^) for 24 h (n=5). **(D)** PC activity in BMDMs isolated from male *ApoE^-/-^* mice fed with a chow diet or HFD (n=5). Data are presented as means ± SD. (B–D) Unpaired two-tailed *t*-test was used. VLDL, very low-density lipoprotein; NS, no significance; FC, fold change; PC, pyruvate carboxylase; BMDM, bone marrow-derived macrophage; HFD, high-fat diet.


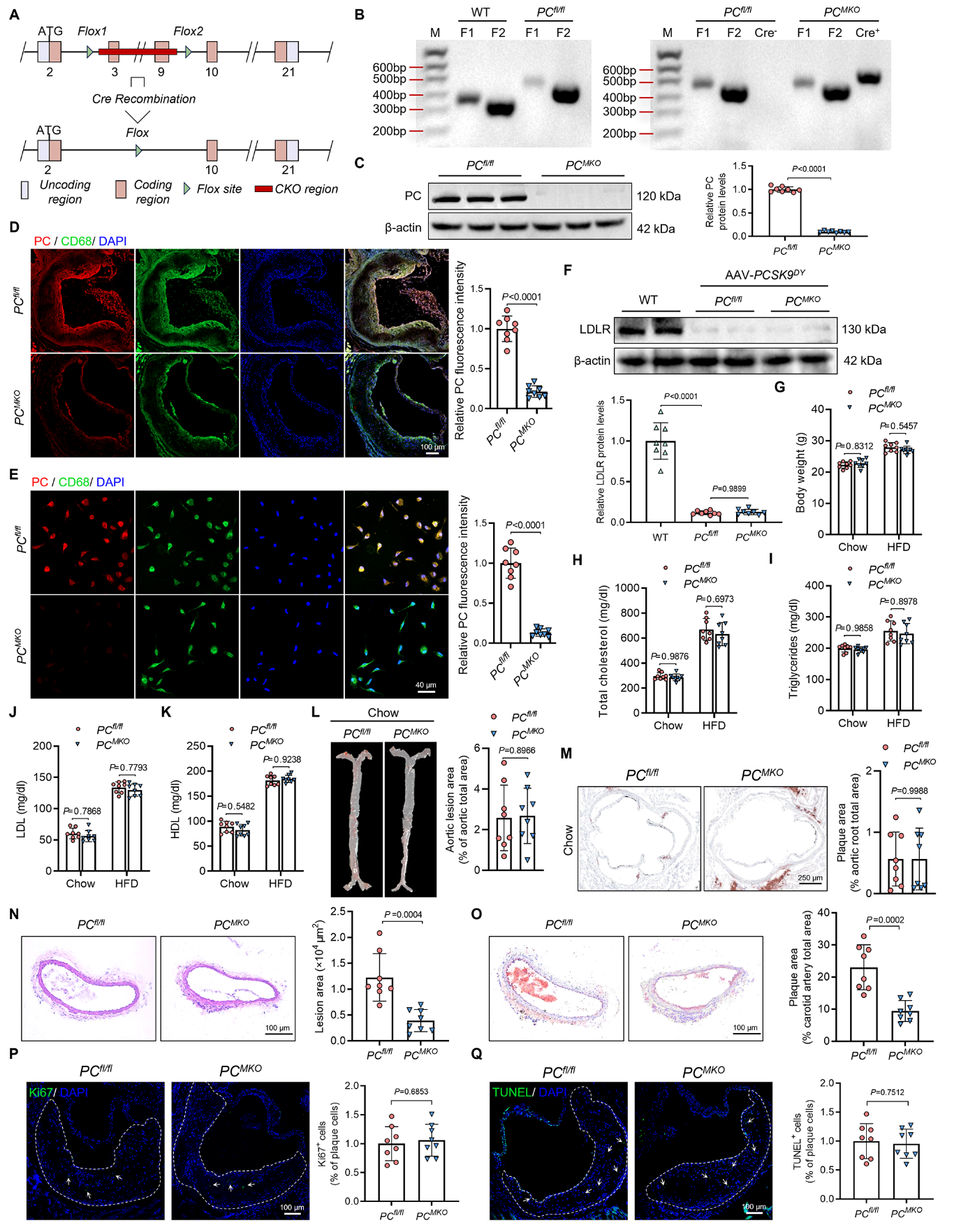


**Figure S2. Identification and atherosclerotic induction of *PC^MKO^* mice. (A)** Construction of *PC^MKO^* mice. **(B)** PCR genotyping using primers specific for flox1 (F1), flox2 (F2), and Lyz2-iCre. Mice carrying F1 and F2 without Lyz2-iCre were referred as *PC^fl/fl^*, whereas mice carrying F1, F2, and Lyz2-iCre were referred as *PC^MKO^*. **(C)** PC protein levels in BMDMs isolated from male *PC^fl/fl^* and *PC^MKO^* mice (n=8). **(D)** Representative immunofluorescence images of PC and CD68 expression in the aortic roots of *PC^fl/fl^* and *PC^MKO^* mice (n=8). Scale bars: 100 μm. **(E)** Representative immunofluorescence images of PC and CD68 expression in BMDMs from *PC^fl/fl^* and *PC^MKO^* mice (n=8). Scale bars: 40 μm. **(F)** Hepatic LDLR protein levels in WT mice and conditional knock-out mice administrated with AAV-*PCSK9^DY^* for 12 weeks (n=8). Body weights **(G)**, total cholesterol **(H)**, triglyceride **(I)**, LDL **(J)**, HDL **(K)** levels of *PC^fl/fl^* and *PC^MKO^* mice administrated with AAV-*PCSK9^DY^* and fed with a chow diet or HFD for 12 weeks (n=8). **(L)** Representative images and quantification of the Oil Red O-stained aortas from *PC^fl/fl^* and *PC^MKO^* mice administered with AAV-*PCSK9^DY^* and fed with a 12-week chow diet (n=8). **(M)** Representative images and quantification of the Oil Red O-stained aortic root sections (n=8). Scale bars: 250 μm. **(N)** Representative images of HE staining in carotid arteries from *PC^fl/fl^* and *PC^MKO^* mice administered with AAV-*PCSK9^DY^* and fed with a 12-week HFD (n=8). **(O)** Representative images and quantification of the Oil Red O-stained carotid arteries from *PC^fl/fl^* and *PC^MKO^* mice administered with AAV-*PCSK9^DY^* and fed with a 12-week HFD (n=8). **(P)** Representative immunofluorescence images and quantification of Ki67 expression in the aortic roots of *PC^fl/fl^* and *PC^MKO^* mice administrated with AAV-*PCSK9^DY^* and fed with HFD for 12 weeks (n=8). Scale bars: 100 μm. **(Q)** Representative immunofluorescence images of TUNEL staining in the aortic roots of *PC^fl/fl^* and *PC^MKO^* mice administrated with AAV-*PCSK9^DY^* and fed with HFD for 12 weeks (n=8). Scale bars: 100 μm. Data are presented as means ± SD. (C-E) and (L-Q) Unpaired 2-tailed *t* test was used. (F) One-way analysis of variance (ANOVA) with Tukey’s correction was used. (G-K) Two-way ANOVA with Tukey’s correction was used. PC, pyruvate carboxylase; WT, wide type; AAV, adeno-associated virus; HFD, high-fat diet; LDL, low-density lipoprotein; HDL, high-density lipoprotein.


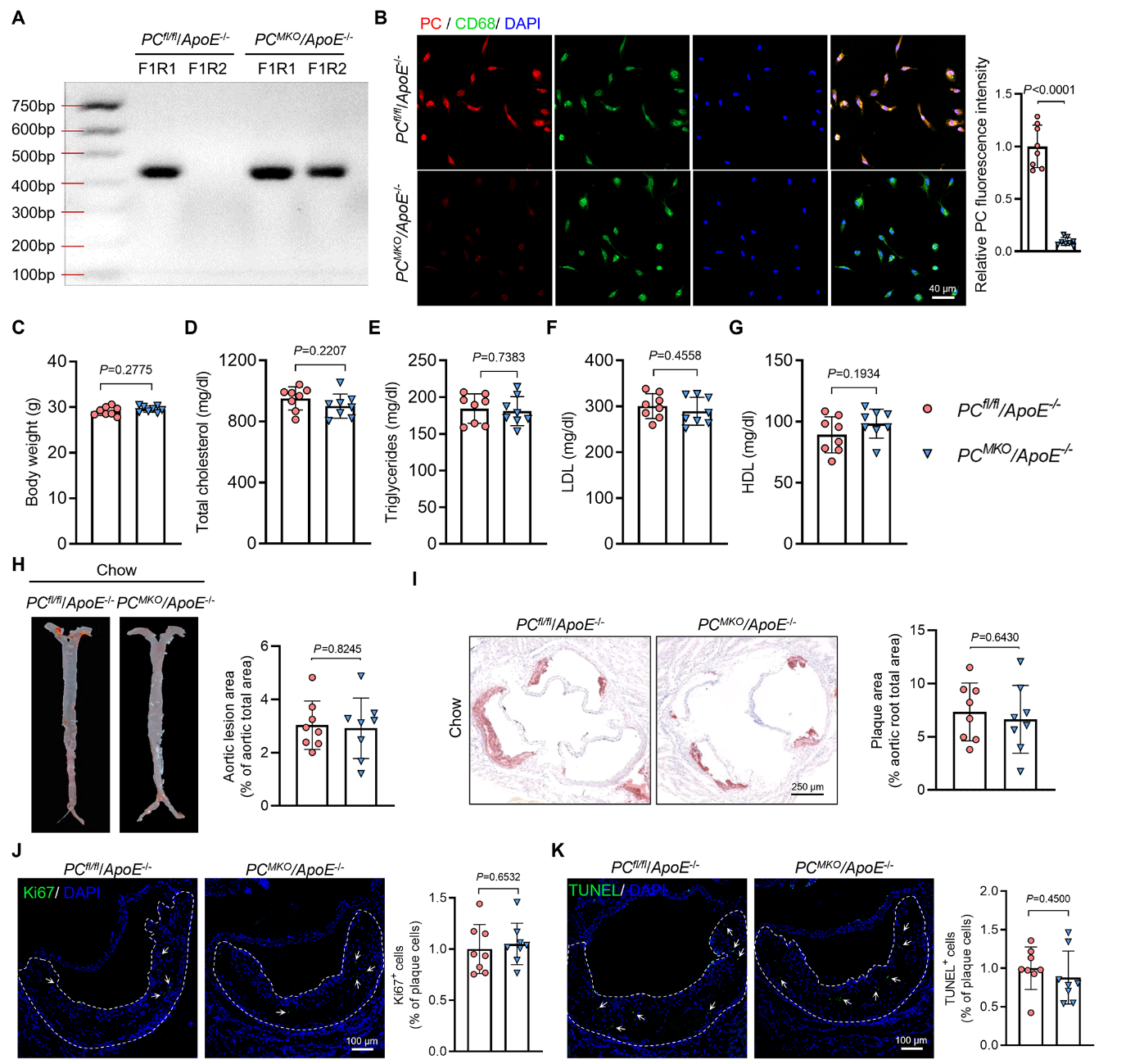


**Figure S3. Identification and atherosclerotic induction of chimaeric *ApoE^-/-^* mice. (A)** PCR analysis for PC alleles in the peripheral blood from irradiated *ApoE^-/-^* mice transplanted with *PC^fl/fl^* and *PC^MKO^* bone marrow. PCR genotyping using primers specific for F1R1 and F1R2. *PC^fl/fl^* Mice carried F1R1, whereas F1R2 decided whether bone marrow cells of *PC^fl/fl^* or *PC^MKO^* were successfully engrafted in *ApoE^-/-^* mice. The PCR products for the *PC^fl/fl^* alleles are 426 bp in length, and the alleles of *PC^fl/fl^/ApoE^-/-^* and *PC^MKO^/ApoE^-/-^* are 6448 bp and 427 bp in length. **(B)** Representative immunofluorescence images of PC and CD68 expression in BMDMs from *PC^fl/fl^/ApoE^-/-^* and *PC^MKO^/ApoE^-/-^* mice (n=8). Scale bars: 40 μm. Body weights **(C)**, total cholesterol **(D)**, triglyceride **(E)**, LDL **(F)**, HDL **(G)** levels of *PC^fl/fl^/ApoE^-/-^* and *PC^MKO^/ApoE^-/-^* mice fed with HFD for 12 weeks (n=8). **(H)** Representative images and quantification of the Oil Red O-stained aortas from *PC^fl/fl^/ApoE^-/-^* and *PC^MKO^/ApoE^-/-^* mice after a 12-week chow diet (n=8). **(I)** Representative images and quantification of the Oil Red O-stained aortic root sections (n=8). Scale bars: 250 μm. **(J)** Representative immunofluorescence images and quantification of Ki67 expression in the aortic roots of *PC^fl/fl^/ApoE^-/-^* and *PC^MKO^/ApoE^-/-^* mice after a 12-week HFD (n=8). Scale bars: 100 μm. **(K)** Representative immunofluorescence images of TUNEL staining in the aortic roots of *PC^fl/fl^/ApoE^-/-^* and *PC^MKO^/ApoE^-/-^* mice after a 12-week HFD (n=8). Scale bars: 100 μm. Data are presented as means ± SD. (B-K) Unpaired 2-tailed *t* test was used. BMDM, bone marrow-derived macrophage; PC, pyruvate carboxylase; HFD, high-fat diet; LDL, low-density lipoprotein; HDL, high-density lipoprotein.


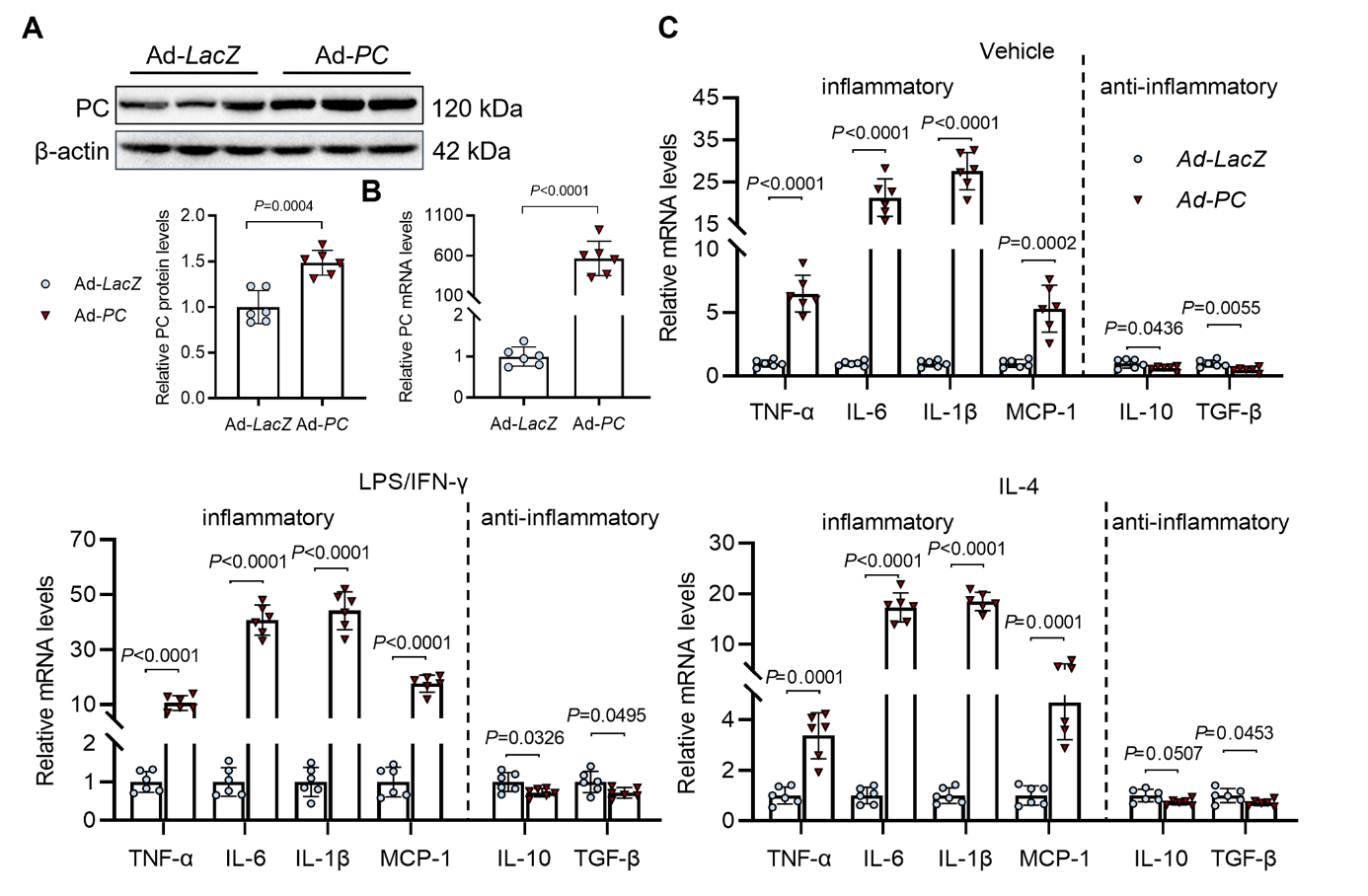


**Figure S4. PC overexpression promoted an inflammatory macrophage phenotype. (A)** Representative PC western blotting and protein quantification in mouse primary BMDMs transfected with Ad-*LacZ* and Ad-*PC* (n=6). **(B)** PC mRNA levels of BMDMs transefected with Ad-*LacZ* and Ad-*PC* (n=6). **(C)** Quantification of selected mRNA in BMDMs transefected with Ad-*LacZ* and Ad-*PC* treated differently (n=6). Data are presented as means ± SD. (A–C) Unpaired 2-tailed *t* test was used. BMDM, bone marrow-derived macrophage; PC, pyruvate carboxylase.


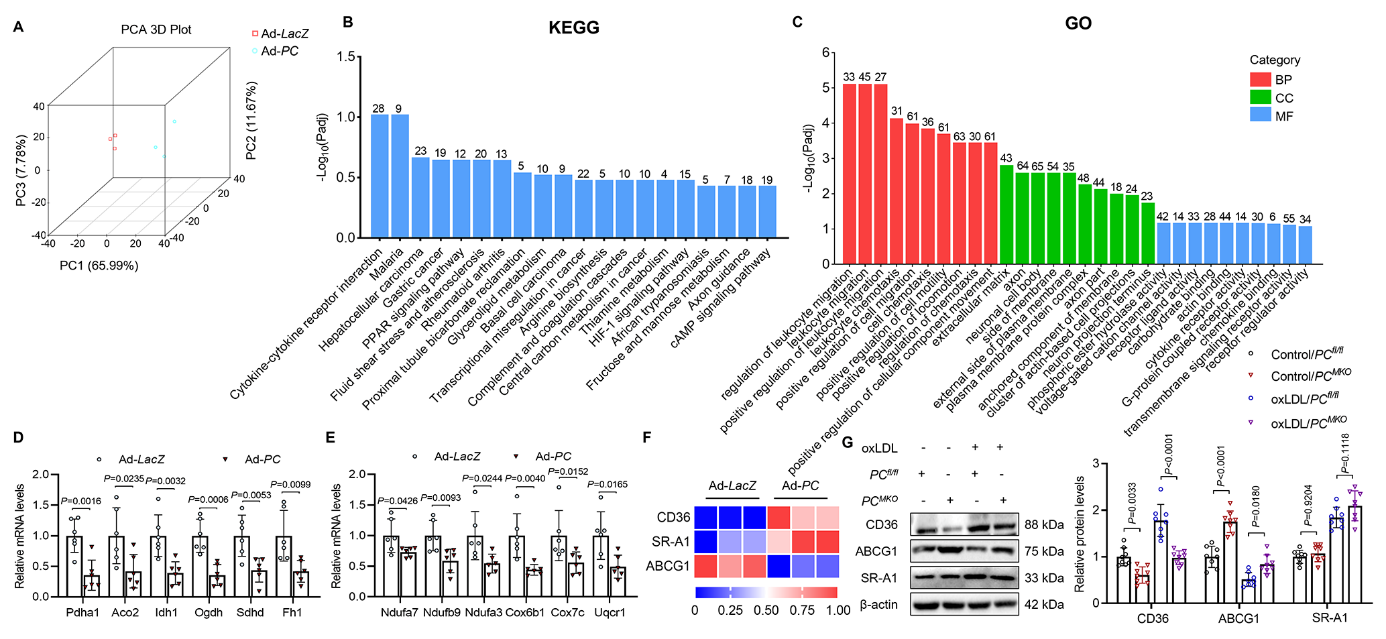


**Figure S5. RNA-sequence profiles and gene expression of mouse BMDMs with PC overexpression or deletion*.*** **(A)** PCA of RNA-sequence results (n=3). **(B)** Top 20 KEGG enrichment of up- and down-regulated expressed genes in BMDMs with PC overexpression. **(C)** Top 10 ranking enrichment of GO biological processes, cell component, and molecular function terms of up- and down-regulated expressed genes in BMDMs with PC overexpression. **(D)** The mRNA levels of enzymes in TCA cycle in BMDMs with PC overexpression (n=6). **(E)** The mRNA levels of genes encoding mitochondrial ETC complexes in BMDMs with PC overexpression (n=6). **(F)** Heatmap showing the expression of scavenger receptors in BMDMs with PC overexpression. **(G)** Expression of scavenger receptors in BMDMs from male *PC^fl/fl^* and *PC^MKO^* mice treated with oxLDL (80 μg mL^-1^) or PBS (n=8). Data are presented as means ± SD. (D) Unpaired 2-tailed *t* test was used. (E) Unpaired 2-tailed *t* test and Mann-Whitney U test with the exact method were used. (G) Two-way analysis of variance with Tukey’s correction was used. BMDM, bone marrow-derived macrophage; PCA, principal component analysis; PC, pyruvate carboxylase; TCA, tricarboxylic acid; ETC, electron transport chain; oxLDL, oxidized low-density lipoproteins.


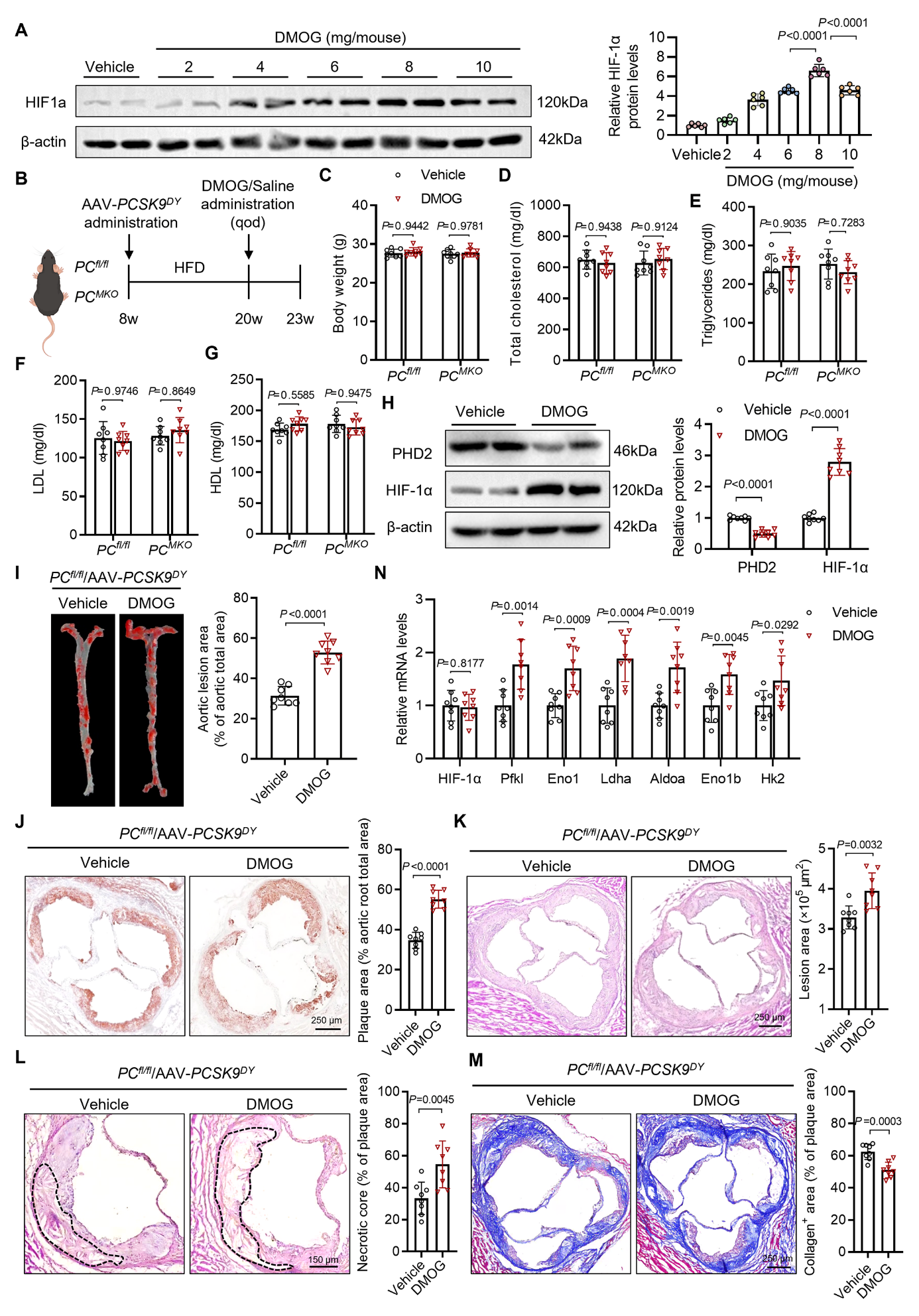


**Figure S6. HIF-1α stabilazetion reversed the protective effect of PC deletion in macrophages on atherosclerosis. (A)** Male *PC^fl/fl^* mice were injected with HIF-1α stabilizer DMOG at different concentration (2/4/6/8/10 mg per mouse) or saline every other day for 3 weeks (n=6). Representative HIF-1α western blotting and protein quantification in *PC^fl/fl^* BMDMs. **(B)** Male *PC^fl/fl^* and *PC^MKO^* mice were administrated with AAV-*PCSK9^DY^* followed by HFD feeding for 12 weeks, and injected with HIF-1α stabilizer DMOG (8 mg per mouse) or saline for the last 3 weeks (n=8). Body weights **(C)**, total cholesterol **(D)**, triglyceride **(E)**, LDL **(F)**, HDL **(G)** levels of *PC^fl/fl^* and *PC^MKO^* mice administrated with AAV-*PCSK9^DY^* and fed with HFD for 12 weeks, with DMOG or saline injection (n=8). **(H)** Representative HIF-1α and PHD2 western blotting and protein quantification in *PC^MKO^* BMDMs (n=8). **(I)** Representative images and quantification of the Oil Red O-stained aortas from *PC^fl/fl^* mice with DMOG or saline injection (n=8). **(J)** Representative images and quantification of the Oil Red O-stained aortic root sections from *PC^fl/fl^* mice with DMOG or saline injection (n=8). Scale bars: 250 μm. **(K-L)** Representative images of HE staining in aortic root sections from *PC^fl/fl^* mice with DMOG or saline injection (n=8). Lesion area **(K)** and necrotic core area **(L)** of aortic root was quantified. Scale bars for (K): 250 μm; Scale bars for (L): 150 μm. **(M)** Representative images and collagen quantification of the aortic roots from *PC^fl/fl^* mice with DMOG or saline injection stained with Masson staining (n=8). Scale bars: 250 μm. **(N)** Quantification of selected mRNA in BMDMs from *PC^MKO^* mice with DMOG or saline injection (n=8). Data are presented as means ± SD. (A) One-way analysis of variance (ANOVA) with Tukey’s correction was used. (C-G) Two-way ANOVA with Tukey’s correction was used. (H-N) Unpaired 2-tailed *t* test was used. PC, pyruvate carboxylase; DMOG, dimethyloxalylglycine; AAV, adeno-associated virus; HFD, high-fat diet; LDL, low-density lipoprotein; HDL, high-density lipoprotein.


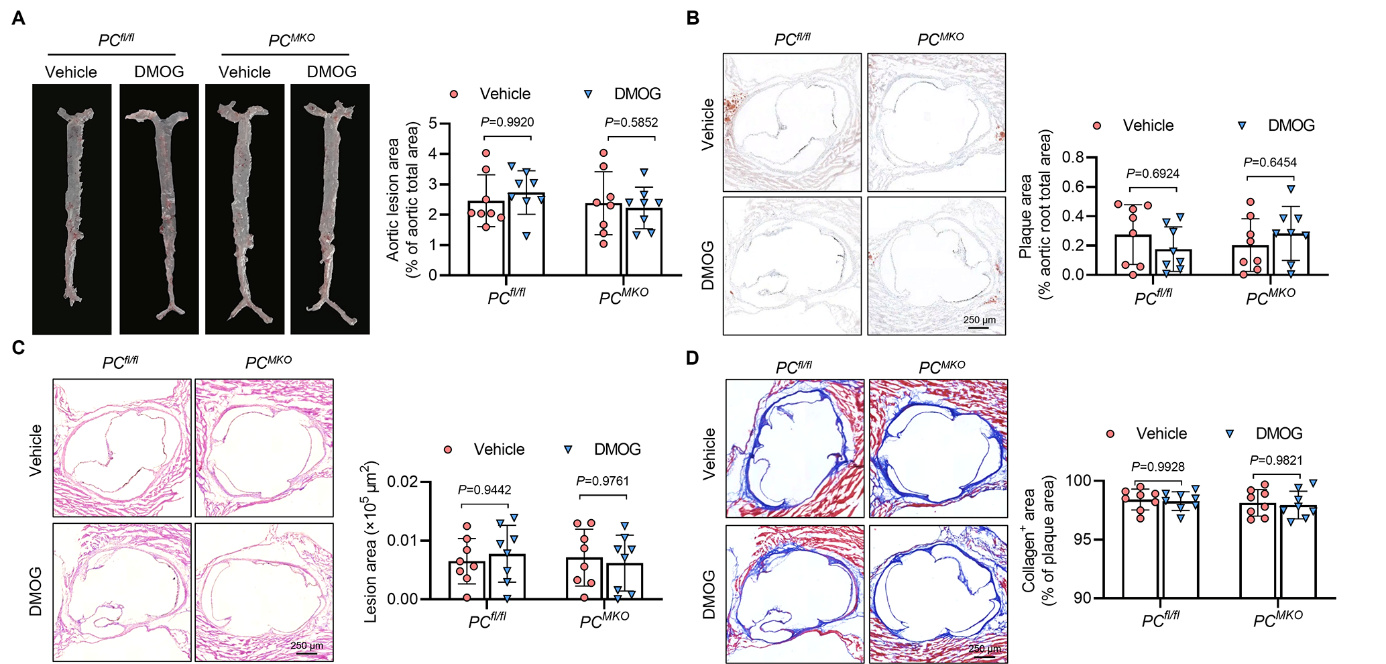


**Figure S7. DMOG did not influence mice without atherosclerotic induction.** (**A-D**) Male *PC^fl/fl^* and *PC^MKO^* mice were administrated with AAV-*PCSK9^DY^* followed by a chow diet feeding for 12 weeks, and injected with HIF-1α stabilizer DMOG (8 mg per mouse) or saline for the last 3 weeks (n=8). **(A)** Representative images and quantification of the Oil Red O-stained aortas from *PC^fl/fl^* and *PC^MKO^* mice with DMOG or saline injection (n=8). **(B)** Representative images and quantification of the Oil Red O-stained aortic root sections from *PC^fl/fl^* and *PC^MKO^* mice with DMOG or saline injection (n=8). Scale bars: 250 μm. **(C)** Representative images quantification of HE staining in aortic root sections from *PC^fl/fl^* and *PC^MKO^* mice with DMOG or saline injection (n=8). Scale bars: 250 μm. **(D)** Representative images and collagen quantification of the aortic roots from *PC^fl/fl^* and *PC^MKO^* mice with DMOG or saline injection stained with Masson staining (n=8). Scale bars: 250 μm. Data are presented as means ± SD. (A-D) Two-way analysis of variance (ANOVA) with Tukey’s correction was used. PC, pyruvate carboxylase; DMOG, dimethyloxalylglycine; AAV, adeno-associated virus.


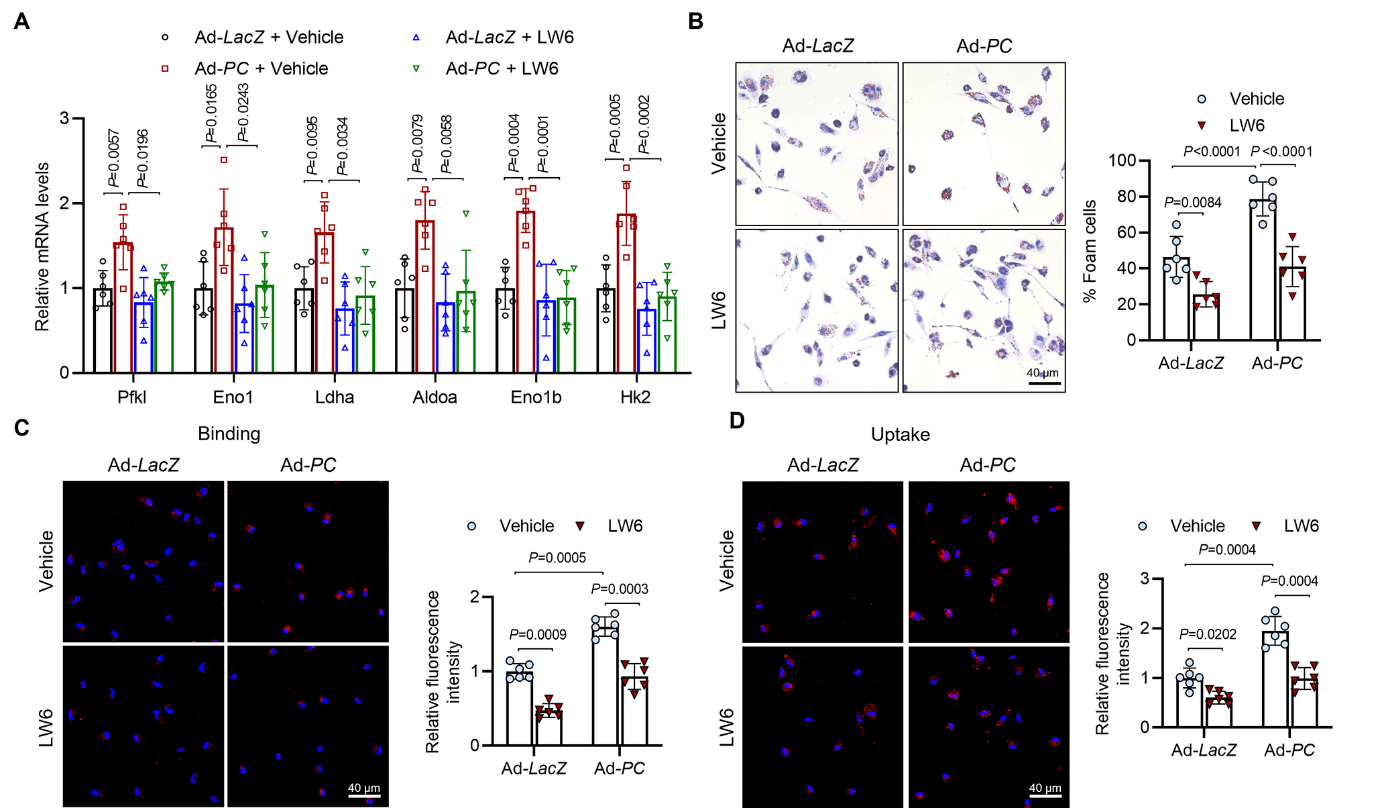


**Figure S8. HIF-1α inhibition prevented foam cell formation promoted by PC overexpression in macrophages. (A)** Mouse primary BMDMs were transfected with Ad-*LacZ* or Ad-*PC*, and treated with LW6 (20 μmol L^-1^) or DMSO for 24 h (n=6). Selected mRNA levels in BMDMs were quantified. **(B-D)** Mouse primary BMDMs were transfected with Ad-*LacZ* or Ad-*PC* and stimulated with oxLDL (80 μg mL^-1^) for 24 h. Then BMDMs were treated with LW6 (20 μmol L^-1^) or DMSO (n=6). **(B)** Representative images of foam cells in male *PC^fl/fl^* and *PC^MKO^* BMDMs. Scale bars: 40 μm. **(C-D)** Representative images of binding **(C)** and uptake **(D)** of Dil labeled oxLDL (Dil-oxLDL) in BMDMs. Scale bars: 40 μm. Data are presented as means ± SD. (A-D) Two-way analysis of variance (ANOVA) with Tukey’s correction was used. BMDM, bone marrow-derived macrophage; PC, pyruvate carboxylase.
