# Supplementary material for: Pyruvate Carboxylase in Macrophages Aggravates Atherosclerosis by Regulating Metabolism Reprogramming to Promote Inflammatory Responses Through the Hypoxia‐Inducible Factor‐1 Signaling Pathway
Source: Adv Sci (Weinh). 2025 May 20;12(29):e17128. doi: 10.1002/advs.202417128 (PMC12362773; doi:10.1002/advs.202417128)
Supplement: Supplementary file 2 — Supporting Information [file ADVS-12-e17128-s002.pdf]

ADVANCED SCIENCE
Open Access

Supporting Information

for *Adv. Sci.*, DOI 10.1002/advs.202417128

Pyruvate Carboxylase in Macrophages Aggravates Atherosclerosis by Regulating Metabolism Reprogramming to Promote Inflammatory Responses Through the Hypoxia-Inducible Factor-1 Signaling Pathway

Ling-Na Zhao, Rui-Ling Wang, Ran-Xin Liu, Meng-Ru Zheng, Li Zhao, Bao-Feng Li, Jia-Le Li, De-Shen Liu, Xiao-Xia He, Qin-Bao Peng, Kai Li, Tian-Xiao Lin, Ying-Ying Liu, Sheng-Ping He, Jun Lu, Shao-Yi Zheng, Xiu Liu* and Fang-Ze Huang**

Original western blots

Figure S2C

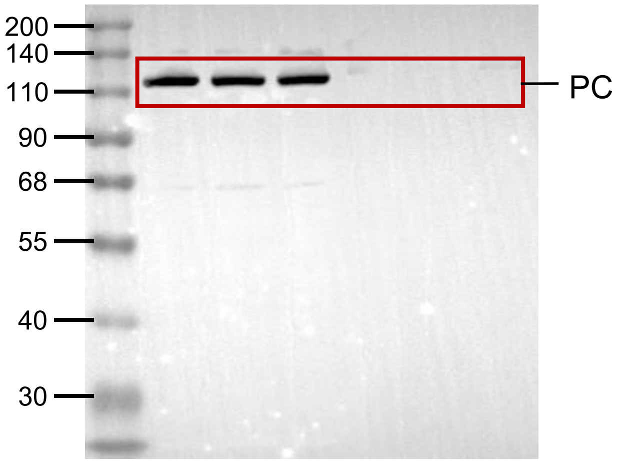

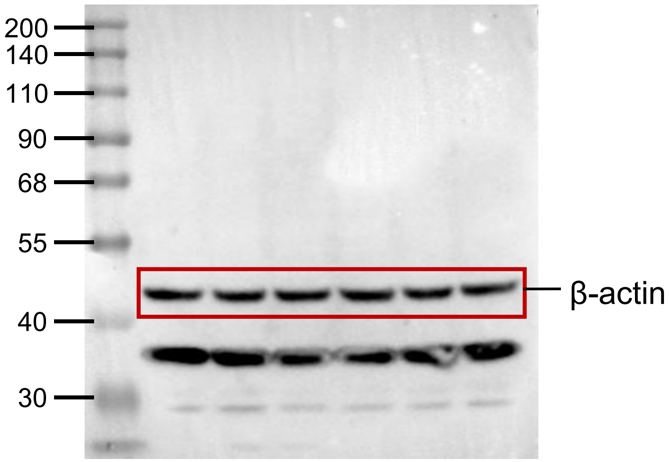

Figure S2F

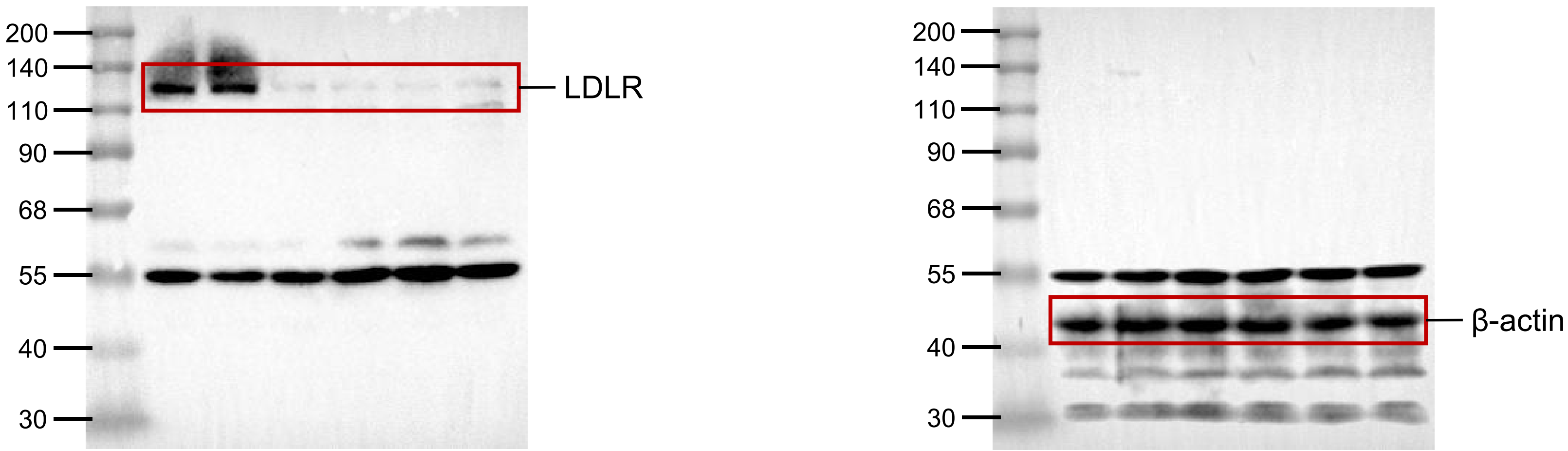

Figure S4A

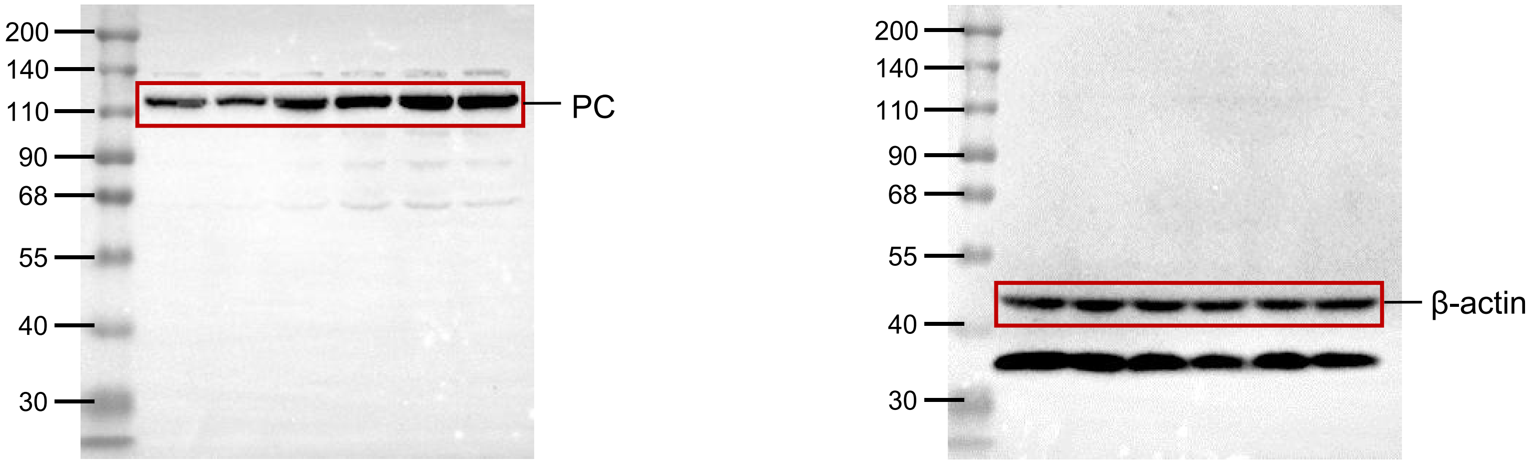

Figure 5H

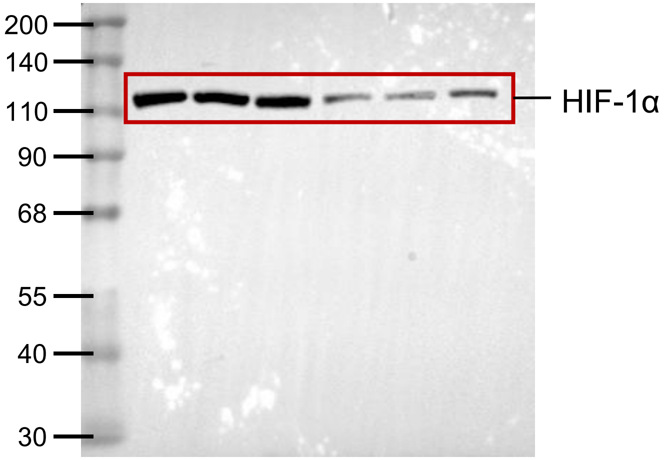

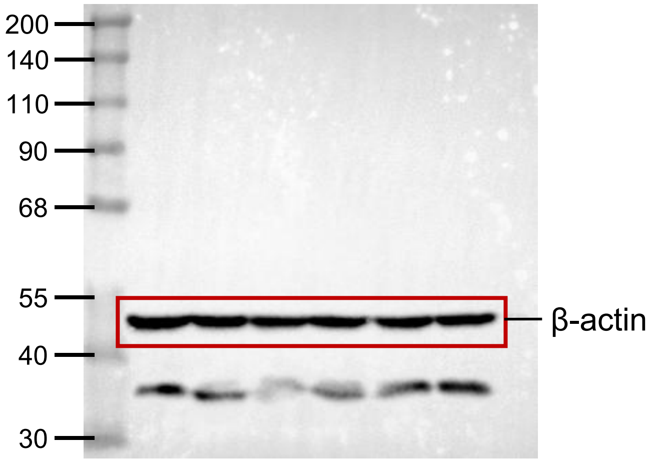

Figure S5G

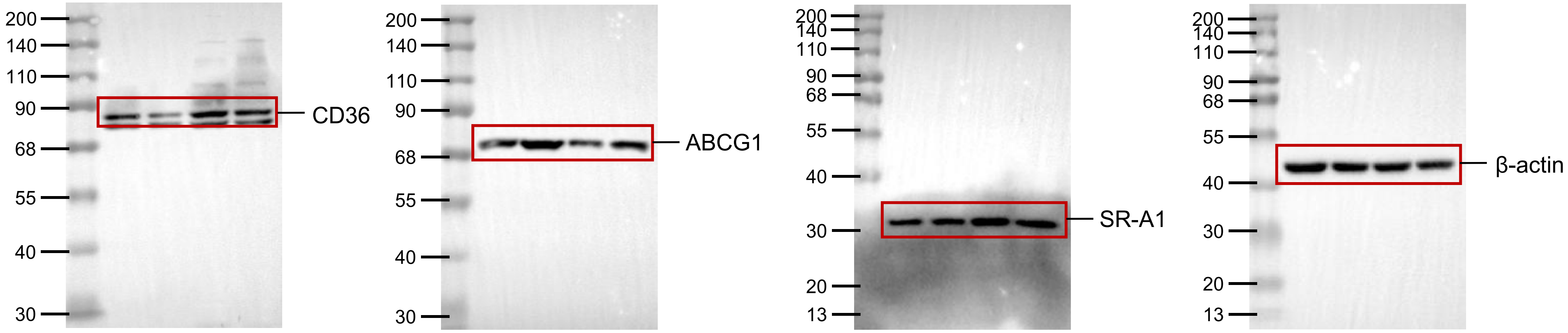
200
140
110
90
68
55
40
30
CD36
200
140
110
90
68
55
40
30
ABCG1
200
140
110
90
68
55
40
30
20
13
SR-A1
200
140
110
90
68
55
40
30
20
13
β-actin

Figure 6A

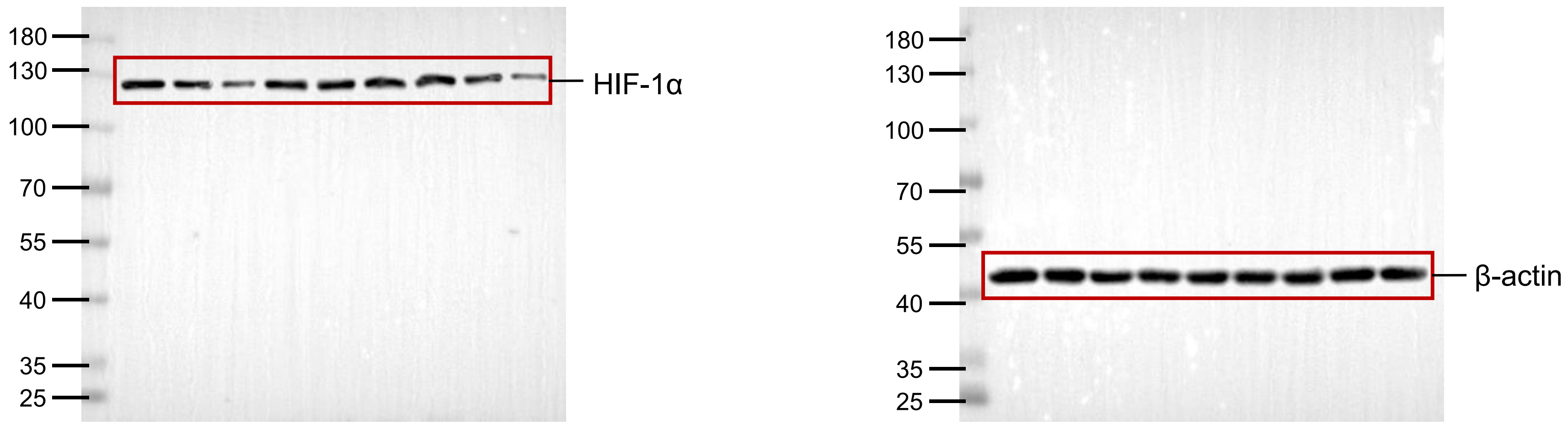

Figure 6C

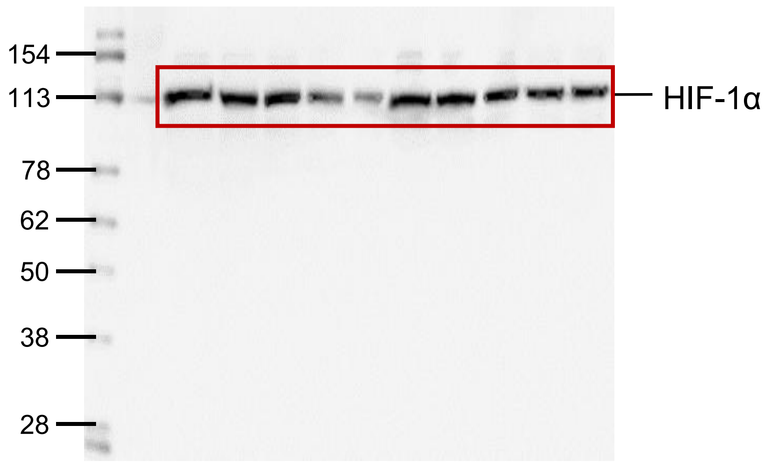

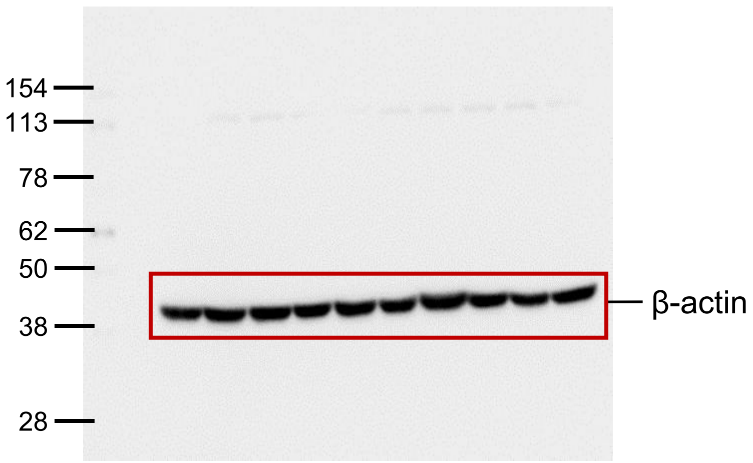

Figure 6E

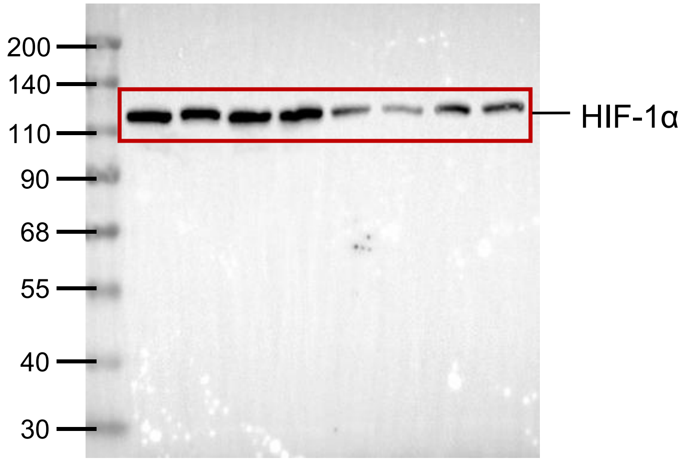

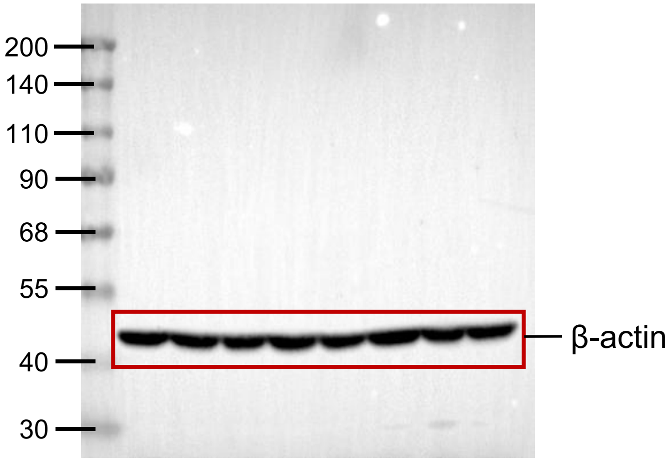

Figure 6G

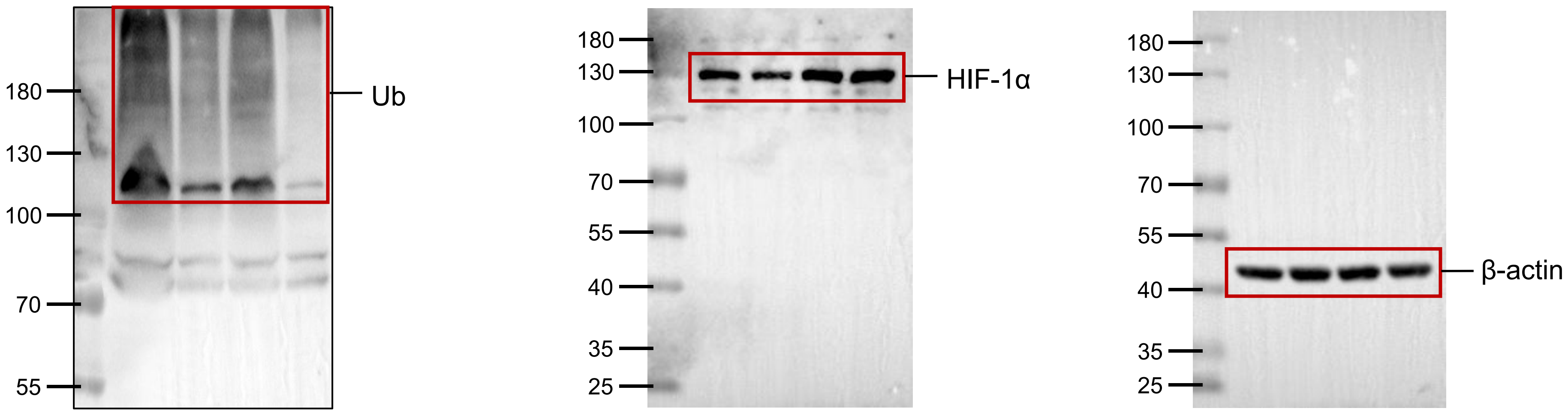

180
130
100
70
55
Ub
180
130
100
70
55
40
35
25
HIF-1α
180
130
100
70
55
40
35
25
β-actin

Figure 6I

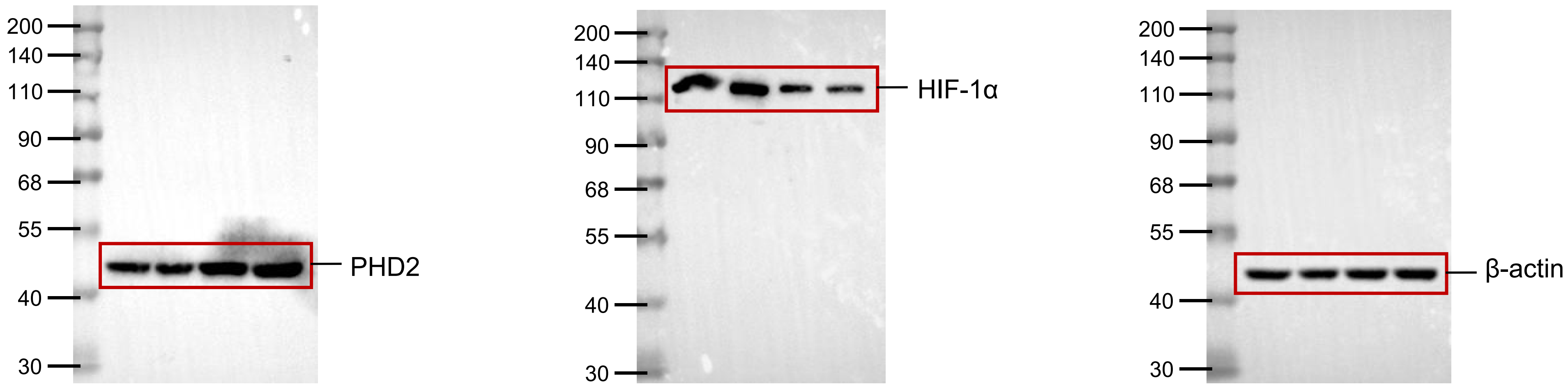

Figure 6K

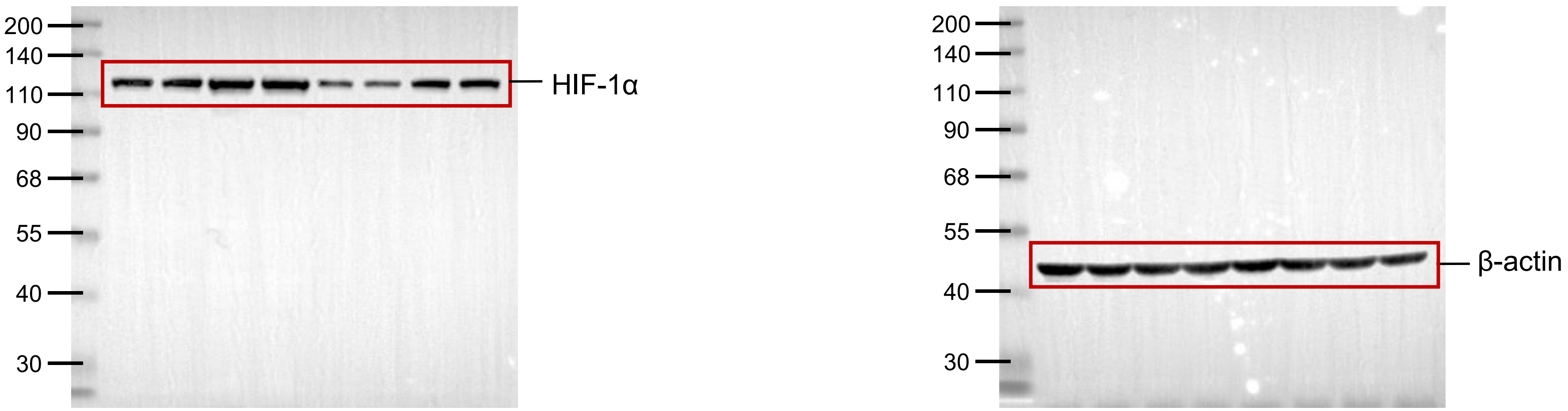

Figure 6M

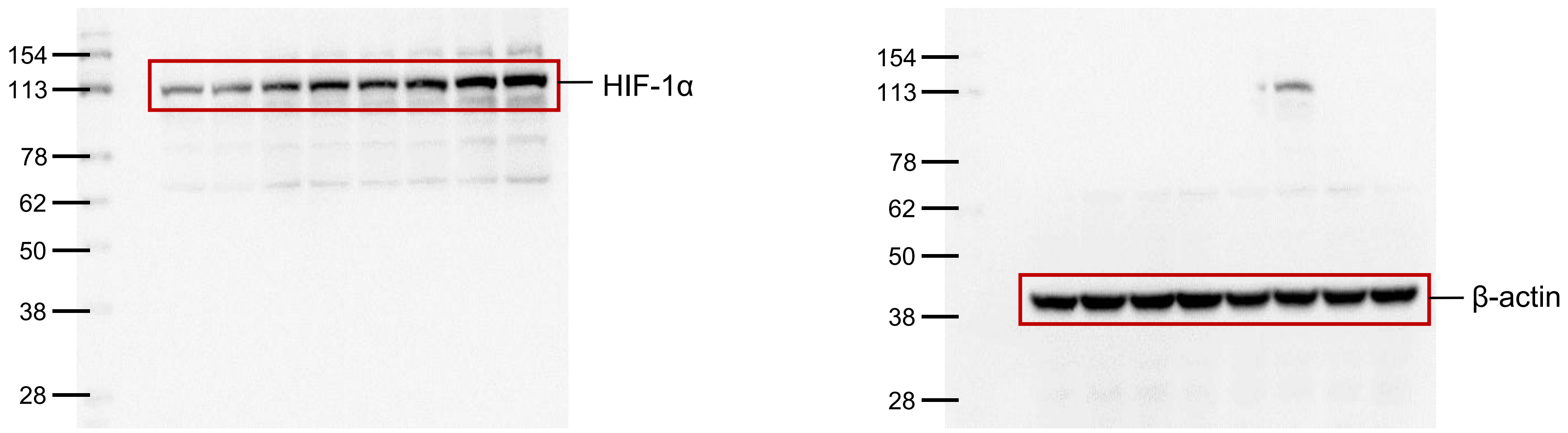

Figure 6O

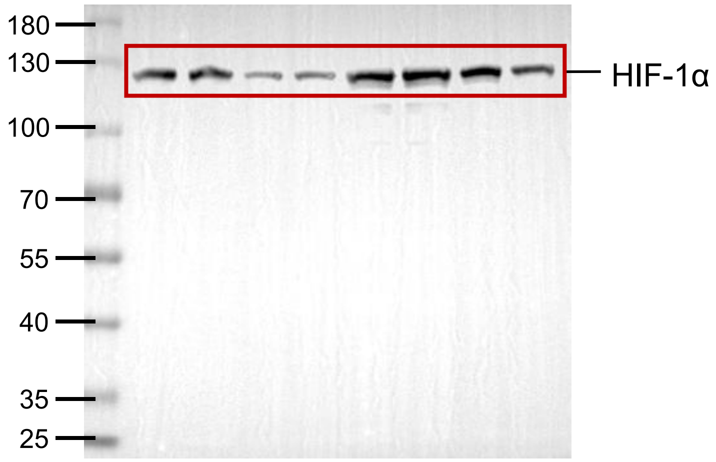

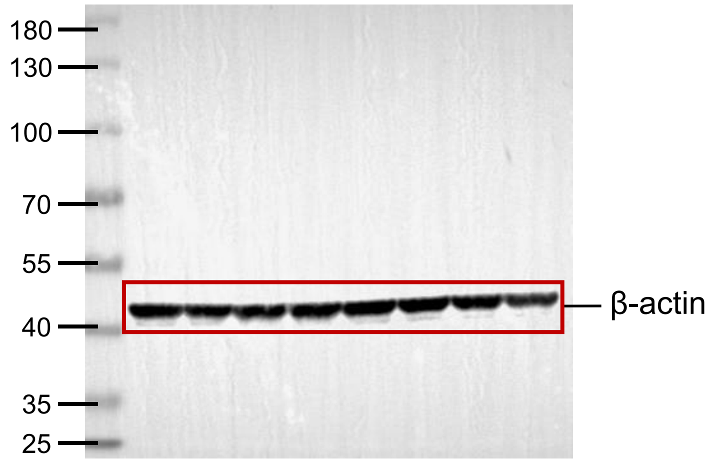

Figure 7C

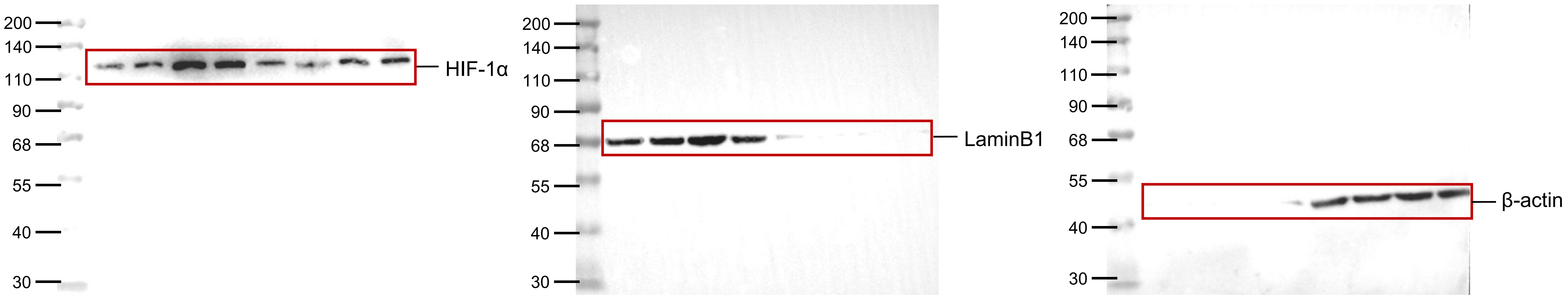

Figure 7J

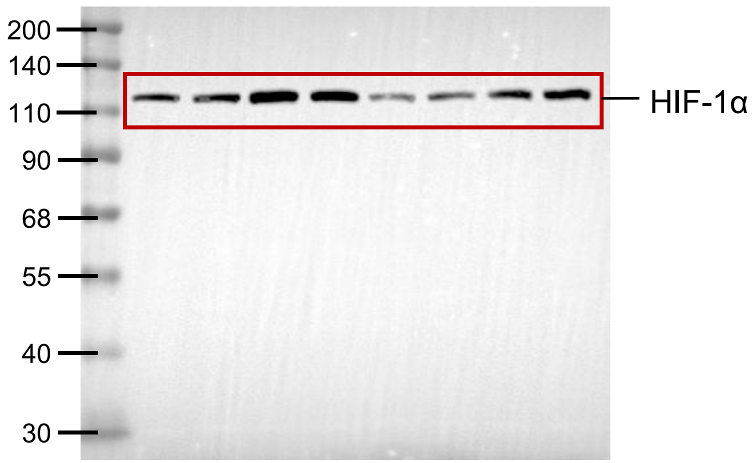

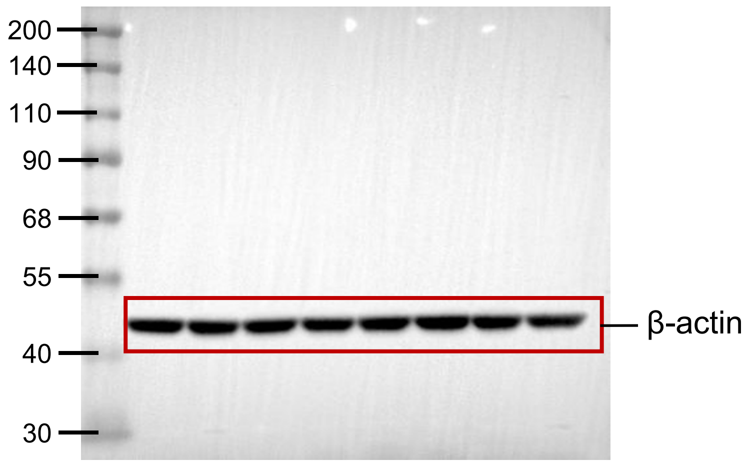

Figure S6A

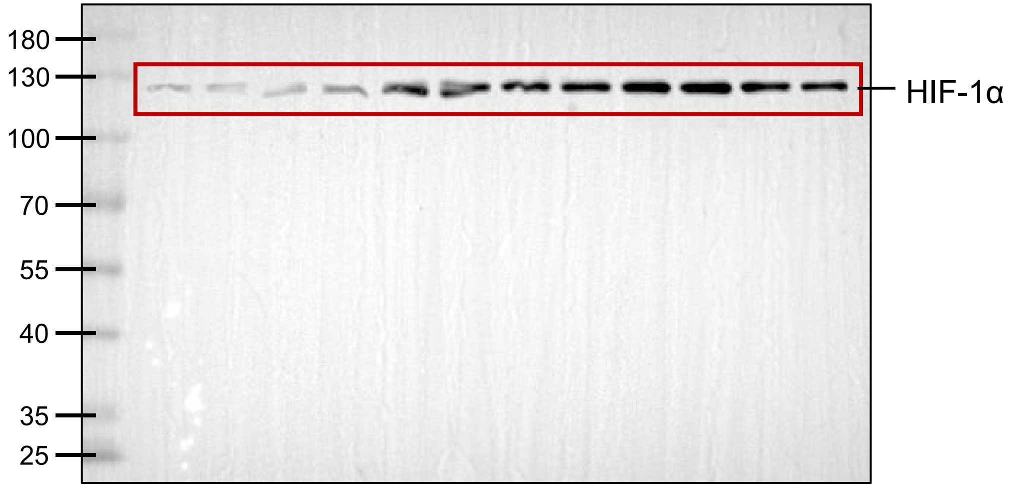

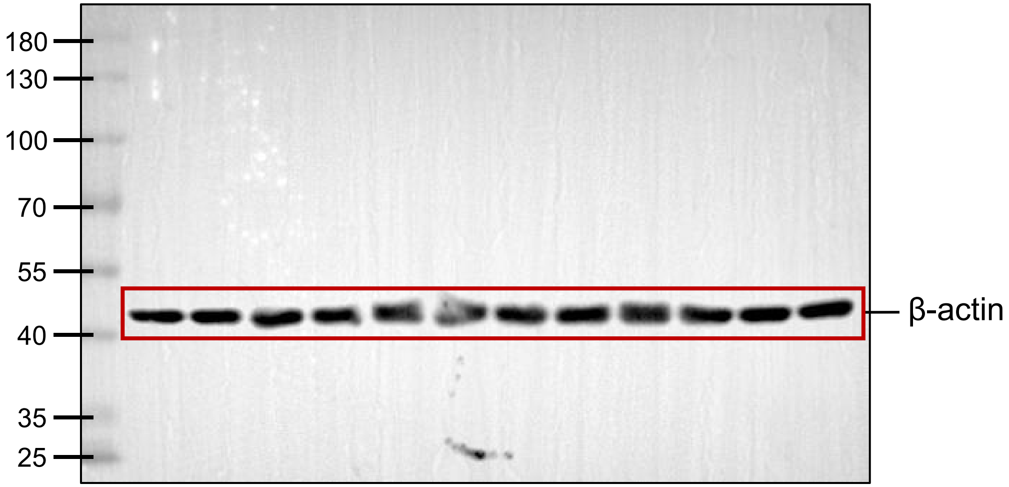

Figure S6H

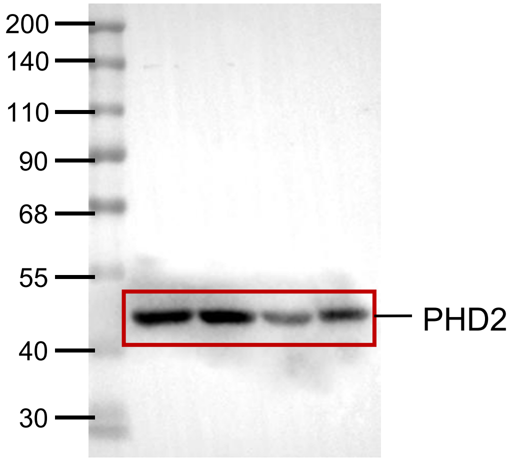
200
140
110
90
68
55
40
30
PHD2

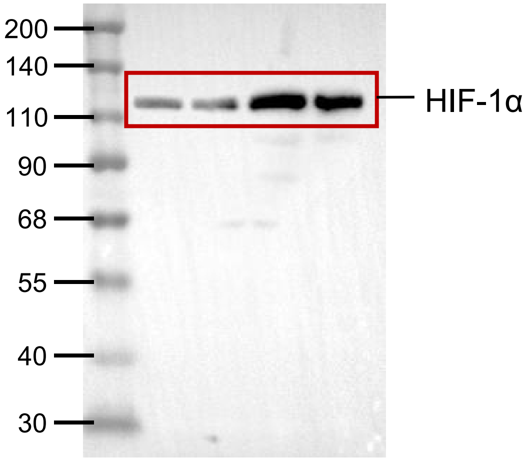
200
140
110
90
68
55
40
30
HIF-1α

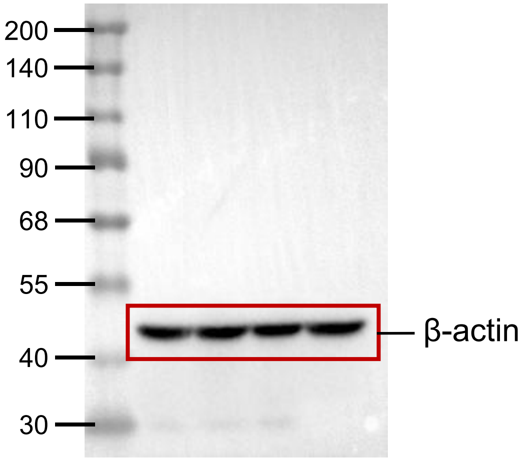
200
140
110
90
68
55
40
30
β-actin
